# Expression and Misexpression of the miR-183 Family in the Developing Hearing Organ of the Chicken

**DOI:** 10.1371/journal.pone.0132796

**Published:** 2015-07-15

**Authors:** Kaidi D. Zhang, Michelle L. Stoller, Donna M. Fekete

**Affiliations:** 1 Department of Biological Sciences, Purdue University, West Lafayette, Indiana, United States of America; 2 Purdue University Center for Cancer Research, Purdue University, West Lafayette, Indiana, United States of America; Harvard University, UNITED STATES

## Abstract

The miR-183 family consists of 3 related microRNAs (miR-183, miR-96, miR-182) that are required to complete maturation of primary sensory cells in the mammalian inner ear. Because the level of these microRNAs is not uniform across hair cell subtypes in the murine cochlea, the question arises as to whether hair cell phenotypes are influenced by microRNA expression levels. To address this, we used the chicken embryo to study expression and misexpression of this gene family. By in situ hybridization, expression of all 3 microRNAs is robust in immature hair cells of both auditory and vestibular organs and is present in the statoacoustic ganglion. The auditory organ, called the basilar papilla, shows a weak radial gradient (highest on the neural side) in prosensory cells near the base on embryonic day 7. About nine days later, the basilar papilla also displays a longitudinal gradient (highest in apical hair cells) for the 3 microRNAs. Tol2-mediated gene delivery was used to ask whether cell phenotypes are malleable when the prosensory epithelium was forced to overexpress the miR-183 family. The expression plasmid included EGFP as a reporter located upstream of an intron carrying the microRNA genes. The vectors were electroporated into the otic cup/vesicle, resulting in strong co-expression of EGFP and the miR-183 family that persisted for at least 2 weeks. This manipulation did not generate ectopic hair cells in non-sensory territories of the cochlear duct, although within the basilar papilla, hair cells were over-represented relative to supporting cells. There was no evidence for a change in hair cell phenotypes, such as short-to-tall, or basal-to-apical hair cell features. Therefore, while increasing expression of the miR-183 family was sufficient to influence cell lineage decisions, it did not redirect the differentiation of hair cells towards alternative radial or longitudinal phenotypes.

## Introduction

The avian basilar papilla (BP) exhibits several similarities to the mammalian hearing organ, the organ of Corti. In both, systematic structural gradients underlie frequency selectivity along the longitudinal axis of the cochlear duct. As one example, the mechanosensory hair cells (HCs) at the base (high frequency; proximal) are shorter and have more stereocilia protruding from their surface than those at the apex (low frequency; distal) [1–3]. A separate functional dichotomy is present across cochlear duct in the orthogonal dimension, called the radial axis. In mammals, the radial axis is notable for its distribution of two major HC types, one row of inner HCs (IHCs) and three rows of outer HCs (OHCs), located on either side of the tunnel of Corti [4]. Likewise in the bird, two distinct populations of HCs are recognized as tall HCs (THCs) on the superior (neural) half of the organ and short HCs (SHCs) on the inferior (abneural) half [5]. Both avian THCs and mammalian IHCs are predominantly innervated by afferents [6, 7], while both avian SHCs and mammalian OHCs receive most of their innervation from efferents and share a functional role in amplifying cochlear vibrations in response to sound [8, 9]. The mechanisms specifying the different cell fates across the radial axis in either birds or mammals is an active area of investigation [10].

The gradation in HC phenotypes along the BP is under the control of at least two important signaling systems. There is an early gradient of BMP7 that is highest at the apex [11]. Subsequently, retinoic acid levels synthesized by Raldh3, which is highest at the apex after embryonic day 10 (E10), can influence stereociliary morphology in a systematic fashion along the BP [12]. Eventually, thousands of genes are differentially expressed in the apex versus the base of the BP at postnatal day 0 (P0) and P14-16 [13, 14]. A subset of the transcripts enriched at the high-frequency end contained predicted binding sites for at least one of 22 different microRNAs (miRNAs) [13]. miRNAs are small non-coding RNAs that down-regulate the expression of target transcripts by binding to their 3’-untranslated regions (3’UTRs) [15–17]. Among the gene sets with miRNA recognition sites statistically over-represented at the high-frequency end were those predicted to bind miR-182 and miR-96. The latter is notable because mutations in the *MIR96* gene underlie inherited hearing loss in both human (DFNA50) and the *Diminuendo* mouse mutant [18, 19]. The 3 members of the miR-183 family (miR-183, miR-96, miR-182) are processed from a single primary transcript [20, 21] and are expressed in sensory cells in mice and zebrafish [20, 22, 23].

A further correlation between the miR-183 family and the tonotopic organization of the mouse cochlea is seen by in situ hybridization. All 3 members of the miR-183 family exhibit dynamic longitudinal gradients postnatally in the mouse organ of Corti [24]. Expression is higher in HCs at the cochlear base at P0 but by P37, this gradient is reversed with higher expression in the apex [24]. These developmental expression profiles led Weston and colleagues [24] to propose that the miR-183 family might enforce reciprocal longitudinal gradients on HC target genes that could underlie, at least in part, the acquisition of phenotypic traits responsible for frequency selectivity. The same study showed that expression of the miR-183 family also varies across the radial axis of the mouse organ of Corti, and that this expression pattern changes with maturation, even as overall levels decrease. At perinatal time points, expression levels are higher in OHCs than IHCs [24, 25]. By P37, this pattern is reversed in the base of the cochlea, while in the apex the levels appear similar across the radial axis, at least as assessed qualitatively from whole mounts [24].

To explore the function of the miR-183 family, expression levels were systematically altered in the developing zebrafish [26]. Zebrafish have HC-bearing sensory organs in both the lateral line and the inner ear. Morpholino-mediated knockdown of the miR-183 family members decreased the number of HCs and otic neurons at 48 hours post fertilization (hpf), while overexpression of miR-96 or miR-182 resulted in ectopic and expanded sensory patches at 26 hpf [26]. These data suggest that the precise level of the miR-183 family members can influence HC specification.

We hypothesize that expression gradients of the miR-183 family reported along and across the mammalian cochlear axis are meaningful for HC development, and thus would be evolutionarily conserved in the bird BP. In this study, we show that the miR-183 family briefly presents with a neural-to-abneural gradient in the chicken BP at E7 and that it also displays an apical-to-basal gradient at E16-18. To evaluate a possible role in establishing or maintaining HC phenotypic gradients in the chicken BP, the miR-183 family was overexpressed in the inner ear prior to and during HC differentiation. Unlike previous results in zebrafish [26], overexpression of the miR-183 family did not reproducibly induce ectopic HCs beyond the sensory domains. With the exception of a mild bias toward the HC fate within the middle regions of the sensory BP, there were no obvious changes in HC or hair bundle morphologies induced by the delivery of the overexpression vector for the miR-183 family.

## Materials and Methods

This study was carried out in accordance with the recommendations of the Public Health Service Policy on Humane Care and Use of Laboratory Animals, from the Office of Laboratory Animal Welfare, National Institutes of Health, which specifically exempts live embryonated eggs (including avian embryos prior to hatching) from the requirement of IACUC approval. The IACUC office at Purdue University granted to D.M.F. a waiver of the animal protocol requirements, thus permitting these experiments to be conducted on chicken embryos.

### Section in situ hybridization

Chicken embryos were removed from the eggs and quickly decapitated. The heads were fixed in 4% paraformaldehyde in phosphate-buffered saline (PBS; pH 7.4) overnight at 4°C and treated with 10%, 20% and 30% sucrose in PBS for cryoprotection. Heads at E5 and E7 were embedded in Tissue Freezing Media (Triangle Biomedical Sciences), or the BPs were isolated and embedded (at E12 and beyond). In situ hybridization was performed on 15 μm horizontal or transverse cryosections as previously described [27]. Briefly, sections were fixed in 4% paraformaldehyde in PBS (10 minutes), digested in 1μg/ml proteinase K (10 minutes), and incubated in triethanolamine and acetic anhydride (10 minutes) followed by hybridization buffer (2 hours). Sections were incubated overnight at 52–56°C in 20nM 3’-digoxygenin-labeled locked nucleic acid (LNA) probes (Exiqon) diluted in hydridization buffer. The LNA probes used were dre/hsa-miR-183, hsa-miR-96 and dre-miR-182. The sections were washed in high salt solutions and rinsed in PBS prior to antibody staining. Antibody blocking solution (10% heat-inactivated goat serum in 100mM Tris pH 7.5 and 0.15M NaCl) was used for one hour and before incubation overnight at 4°C in sheep polyclonal IgG anti-digoxygenin-alkaline phosphatase Fab fragments (Roche 11093274910, 1:2000) diluted in blocking solution. After extensive washes, the sections were incubated with 330μg/ml NBT and 160μg/ml BCIP (Roche) for color development. The color reaction was stopped by washing in 1mM EDTA in PBS. The sections were dehydrated through graded ethanols, cleared with Hemo-De (Scientific Safety Solvents) and coverslipped with ShurMount (Triangle Biomedical Sciences).

### Whole-mount in situ hybridization

E12-E18 chicken embryo heads were immersion-fixed in 4% paraformaldehyde in PBS overnight at 4°C after the removal of the tympanic membranes and the columellas to expose the oval windows. After fixation, the embryonic cochlear duct was dissected from the surrounding temporal bone and the auditory nerve. The abneural nonsensory cells, tegmentum vasculosum and tectorial membrane were removed to isolate the BP. Samples were dehydrated through graded methanols and were stored at -20°C. Whole-mount in situ hybridization was performed as previously described [26, 28]. Briefly, after rehydration in graded methanols, the BPs were treated with 5μg/ml proteinase K for 7 minutes and fixed in 4% paraformaldehyde in PBS for 20 minutes. They were pre-incubated in hybridization buffer for 2–3 hours and then hybridized with 20nM digoxygenin-labeled LNA probes overnight at 52–56°C. After high-stringency washes, digoxygenin-labeled probes were detected by immunostaining and alkaline phosphatase histochemistry as described above for section in situs. Specimens were mounted in glycerol or Vectashield mounting medium (Vector Laboratories).

### Plasmid construction

Plasmid pCAG-T2TP (abbreviated pT2TP) encoding Tol2 transposase and plasmid pT2K-CAG-EGFP (abbreviated pGFP) [29] with flanking Tol2 sites were kindly provided by Yoshiko Takahashi (University of Tokyo, Japan). pGFP was first modified to create a Gateway destination vector (pT2K-CAG-EGFP-attR) by inserting the attR cassette (Invitrogen) into the EcoRV site located after the EGFP coding sequence. An approximately 800 base pair fragment containing genomic sequences from the mouse miR-183 family locus, flanked by splice donor and acceptor sites, was obtained from pME-MCS-sd-miR183F-sa [30]. The miR-183 intron was inserted into pT2K-CAG-EGFP-attR through a Gateway LR recombination reaction, creating pT2K-CAG-EGFP-183F (abbreviated pGFP-183F). Plasmid pT2K-CAG-EGFP-9 (abbreviated pGFP-9) was created by inserting miR-9 coding sequence from pME-MCS-sd-9-sa [30] into pT2K-CAG-EGFP-attR and was used as a control in luciferase assays.

S1 Fig shows a comparison of the miR-183 family mature sequences among human (hsa), mouse (mmu), chicken (gga) and zebrafish (dre). *Gallus gallus* mature miR-183 (gga-miR-183) sequence was obtained from miRBase while the miR-182 and miR-96 sequences were obtained from published *Gallus gallus* short RNA sequencing reads [31]. The sequence of miR-96 is fully conserved between these species, while miR-182 and miR-183 differ by 1–3 nucleotides at their 3’ ends, but are otherwise identical. Across all four species, the seed regions of each family member are perfectly conserved; hence, the miRNA overexpression constructs, designed using mouse genomic sequences, are expected to bind well to consensus sequences on chicken target genes.

### Northern blots

HEK 293T/17 cells (abbreviated HEK293T cells; ATCC #CRL-11268) seeded in 35mm plates were transfected with 2 μg of either pGFP-183F or pGFP using Lipofectamine 2000 (Invitrogen) according to the manufacturer’s instructions. Small RNAs were harvested approximately 30 hours later using the Purelink miRNA Isolation Kit (Invitrogen). Samples (600ng of small RNA) were subsequently probed for miR-183 family expression using DNA probes against the mature miRNA human sequences (Signosis) in conjunction with a chemiluminescence system, the High Sensitive miRNA Northern Blot Assay Kit (Signosis), according to the manufacturer’s instructions.

### miRNA luciferase assays

UMNSAH-DF1 cells (abbreviated DF-1 cells; ATTC #CRL-12203) were seeded in 6-well plates (Costar) 20–24 hours prior to transfection in DF-1 growth medium containing 10% fetal calf serum (Atlanta Biologicals), 2% chicken serum (Sigma) in DMEM supplemented with L-glutamate and penicillin-streptomycin. Immediately before transfection, growth medium was replaced with transfection medium: 10% fetal calf serum in Optimem with Glutamax (Gibco). Plasmids (miRNA reporters and pGFP, pGFP-183F, or pGFP-9) were transfected using Lipofectamine 2000. Details regarding construction of miRNA reporters and the luciferase assays are published [30]. After 24 hours the cells were lysed and luciferase activity was assessed using the dual luciferase assay kit (Promega) in concert with the Luminoskan Ascent luminometer (Thermo Fisher). These experimental values were referenced to the control values (arbitrarily set to one). Each treatment condition was conducted at least in duplicate, and each experiment was repeated at least three times.

### Electroporation of otic cup or otic vesicle

Fertilized White Leghorn chicken eggs (Animal Sciences Research and Education Center, Purdue University) were incubated at 38°C in a humidified incubator and the embryos assigned Hamburger and Hamilton stages (S) [32]. The eggs were windowed at E2 and plasmids were electroporated into the otic cup at S11–S12 or into the otocyst at S14–S17. The chorion and amnion were opened to expose embryo heads. Pulled-glass capillary micropipettes with 10–12 μm tips were loaded with concentrated plasmids (3–5 μg/μl) diluted with 1/10 volume of 0.25% fast green dye. All *in ovo* injections were done with a mixture of the Tol2 transposase vector pT2TP and either pGFP or pGFP-183F. The molar ratio of pT2TP to the GFP plasmids was in the range of 0.75 to 1. The right otic cup or otocyst was injected using a Picospritzer II (Parker Hannifin Corp.). After injection, electroporation was done using a TSS20 ovodyne electroporator and an EP21 current amplifier (Intracel). The electroporation condition was 10V, 2 pulses, 50ms width, and 10ms space between the two pulses. The BP primordium originates from the mid-posterior otic placode [33, 34] and from approximately the ventromedial region of the otocyst [35], so these areas were targeted. For S11–S12 embryos with their dorsal sides facing up, two platinum wires [36] were placed on either side of the embryo at the level of the otic cup, as previously described [37]. For S14–S17 embryos with their right sides facing up, a large opening was made in the amnion so that the negative electrode could be placed above the right otocyst and the positive electrode pushed beneath the left otocyst. A total of 896 embryos received pGFP-183F and 325 embryos received pGFP. Embryos were sacrificed 2–14 days after electroporation. The majority of embryos were intended for analysis 2 weeks after electroporation, although few survived to this time point, regardless of which plasmid was used (13% survival for pGFP and 11% survival for pGFP-183F). Electroporated embryos that were sacrificed at E14-E16 were routinely 1–3 days younger in their Hamburger and Hamilton developmental stages than expected.

### Immunofluorescence

For whole-mount immunostaining, BPs were dissected after overnight immersion in cold 4% paraformaldehyde. The E12-E16 BPs were rinsed with PBS/0.1% triton for 30 minutes and incubated in blocking solution (10% goat or horse serum, 0.1% triton, 0.05% sodium azide in PBS) for at least one hour. They were incubated in primary antibodies overnight at 4°C. Primary antibodies used were mouse monoclonal IgG_2a_ anti-otoferlin HCS-1 (Developmental Studies Hybridoma Bank, 1:200), rabbit polyclonal IgG anti-GFP (Life Technologies A11122, 1:5000), mouse monoclonal IgG_1_ anti-CtBP2 (BD Transduction Labs 612044, 1:300) and goat polyclonal IgG anti-Sox2 (Santa Cruz Biotechnology, 1:500). After extensive washes in PBS/0.1% triton, samples were incubated in AlexaFluor-conjugated secondary antibodies (Life Technologies, 1:500) with phalloidin (Invitrogen, 1:500) or the nuclear dye TO-PRO-3 (Invitrogen, 1:5000) overnight at 4°C. All antibodies were diluted in blocking solution. Specimens were then rinsed and mounted in Vectashield hardset mounting medium (Vector Laboratories).

For section immunostaining, 15 μm cryostat sections from E5-E7 chicken embryos were mounted on slides, post-fixed in 4% paraformaldehyde/PBS for 10 minutes, rinsed with PBS, and blocked in antibody blocking solution (10% goat serum, 0.05% triton, 0.05% sodium azide in PBS). Primary antibodies (HCS-1 and anti-GFP) were incubated for at least 1 hour at room temperature or overnight at 4°C. After PBS washes, the sections were incubated with appropriate AlexaFluor-conjugated secondary antibodies for at least 1 hour, rinsed and coverslipped with Vectashield hardset mounting medium.

### Microscopy and image analysis

Images of immunostained sections and in situ specimens were taken with a Nikon Eclipse 800 equipped with a SPOT Flex digital camera (Diagnostics Instruments) and Spot 5.1 software. When appropriate, images of sections through the left ear and images of whole-mount left BPs were mirror-image flipped to facilitate comparisons with the right ear or the right BPs. Images of immunostained whole-mount BPs were taken with a Nikon C1-plus confocal microscope and a 60X lens. Image stacks were taken at the base (~25%), middle (~50%) and apex (~75%) along the BP (base was defined as 0% and apex 100%). For each HC in the field, the maximal cell perimeter was traced from the HCS-1 red channel without viewing the GFP fluorescence in the green channel, and the cross-sectional areas were calculated using ImageJ. Then each HC was viewed in the green channel and was categorized as either GFP-positive (GFP+) or GFP-negative for separate analysis. The number of ribbons per HC was counted as the number of CtBP2-positive foci. The numbers of GFP+ HCs and GFP+ supporting cells (SC) were counted in confocal image stacks using ImageJ and the ratios of GFP+ SC/HC were calculated. Student t-test and 2-way ANOVA was performed and graphs were made in Prism 6 software.

## Results and Discussion

### Inner ear expression of the miR-183 family at E5 and E7

**Fig 1:**
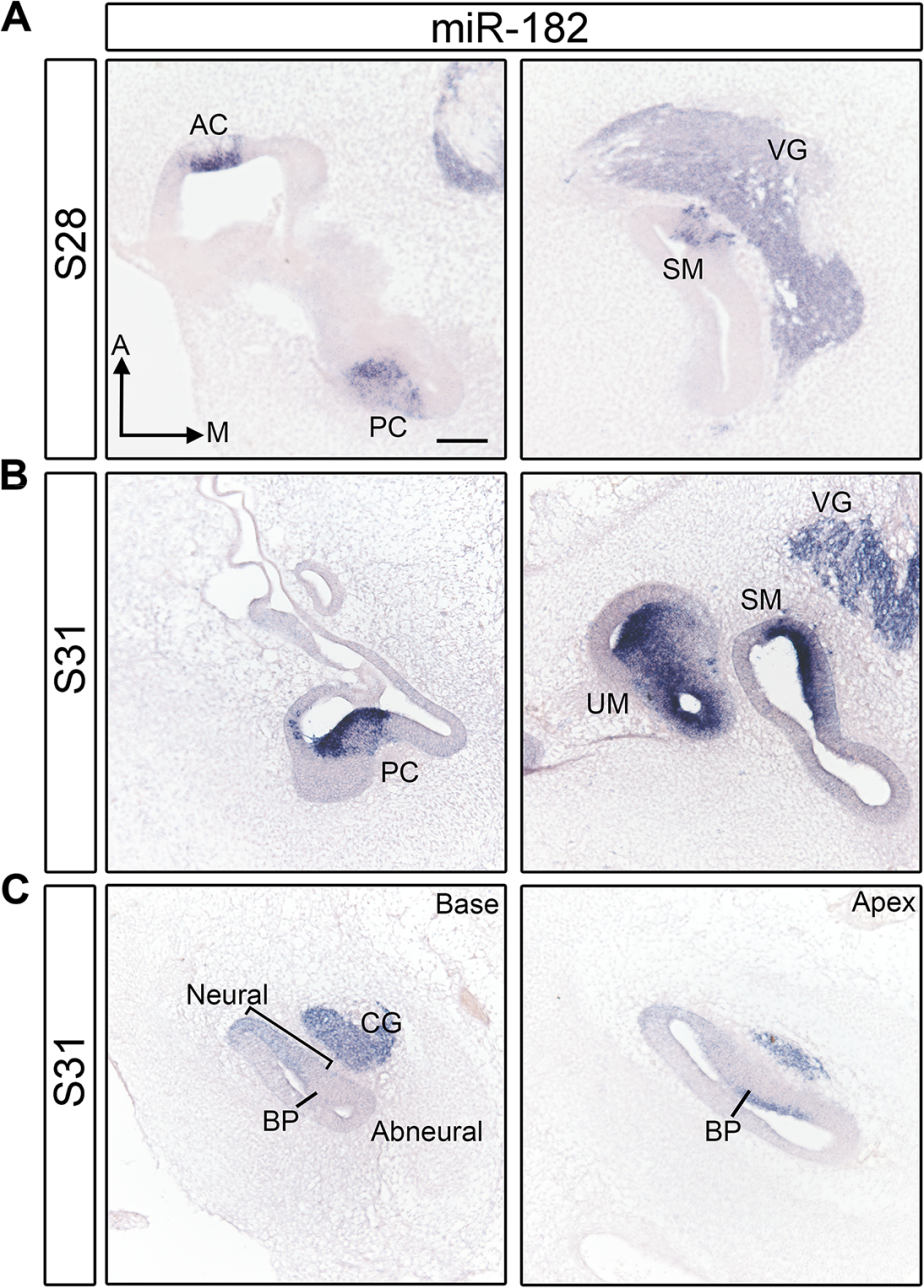
miR-182 is expressed in HCs and ganglion neurons of inner ear at S28 and S31. (A) Section in situ hybridization of miR-182 shows its presence in cristae and maculae at S28. (B) The expression of miR-182 in the HCs of vestibular organs at S31. C: The expression of miR-182 in the cochlear duct at S31. Sections across the base and the apex of the BP are shown. Note that HCs start to differentiate in the apex at this stage. A, anterior; AC, anterior crista; BP, basilar papilla; CG: cochleolagenar ganglion; M, medial; PC, posterior crista; SM, saccular macula; UM, utricular macula; VG, vestibular ganglion. Scale bar equals 100μm.

In situ hybridization was used to detect the expression of mature miR-183, miR-96 and miR-182 on sections through the embryonic chicken inner ear at stages when nascent HCs are present in vestibular and auditory sensory organs (E5/S28 and E7/S31, respectively). Alternate sections were immunostained for otoferlin (using the HCS-1 antibody, S4 Fig) [38]. At both stages, the miR-183 family members were detected in the statoacoustic ganglion neurons and in vestibular HCs of the anterior crista, posterior crista, lateral crista, utricular macula and saccular macula (Fig 1A–1C, S2 and S3 Figs). The miRNAs were undetectable in the BP and lagena macula anlagen at S28, but they were present in both organs at S31 (Fig 1C, S2 and S3 Figs). The detection of the miR-183 family members in both immature HCs and in statoacoustic neurons is similar to that previously described for both mouse [24, 25] and zebrafish [26].

In the vestibular organs at S28 and S31, the strongest hybridization signals were adjacent to the lumenal surface of the epithelium (Fig 1A and 1B, S2 and S3 Figs), which is where the HC nuclei begin to align as the cells become post-mitotic and start to differentiate. We interpret this lumenal signal to reflect expression in nascent HCs. We also observed weaker signals immediately below the HC layer, where SC nuclei are found. The SC cytoplasm stretches from the lumen to the basal lamina of the sensory domains, raising the possibility that these cells could be weakly labeled by the miRNA probes. However, young HCs also project cytoplasmic tails downward toward the basal surface, as observed by HCS-1 immunostaining in alternate sections (arrows in S4 Fig) and reported previously [38]. We interpret weak hybridization signal for the miR-183 family in developing sensory domains as consistent with the presence of thin cytoplasmic tails of HCs within the SC layer, although we cannot definitely exclude expression in prosensory cells or SCs of the vestibular organs at these immature stages.

We were specifically interested in whether inner ear sensory organs showed evidence of the miR-183 family expression at the prosensory stages, or whether expression was only limited to HCs. Both miR-182 and miR-183 were reported to be broadly expressed in the E9.5 mouse otocyst, and present in both prosensory tissues and non-sensory domains in the E12.5 mouse cochlea [25]. However, the expression of the miR-183 family in zebrafish inner ear was not detected in prosensory cells, but was only found in HCs [39]. From our analysis in the avian inner ear, the most definitive evidence for prosensory expression was obtained from the BPs. At S28 when HCs in the basal (proximal) BP start to exit the cell cycle [40], there was no detectable expression of the miRNA-183 family anywhere along its length (n = 3 embryos; S2 and S3 Figs). At S31-32, faint expression of miR-182 was evident in and adjacent to the prosensory domain at the base (proximally), but only on the neural side (n = 7/7 ears from 5 embryos). The signal appeared as a radial gradient, getting progressively weaker from neural-to-abneural (Fig 1C bracket). A similar gradient was evident in adjacent sections probed for miR-183 (n = 3/4 ears from 2 embryos) or miR-96 (n = 2/5 ears from 3 embryos, S3 Fig bracket). In the apical (distal) BP at the same stage, all three members of the miR-183 family were detected in nascent HCs (Fig 1C for miR-182; S2 and S3 Figs). Thus, the miR-183 family belongs to a small group of genes reported as being differentially expressed across the radial axis of the BP as early as E7, when the proximal organ, while postmitotic, is still at the prosensory stage. This includes a subset of Wnt-related ligands, inhibitors and receptors [41].

The sporadic detection of a radial expression gradient in the prosensory BP deserves further comment. In the mouse, all 3 miR-183 family members are derived from a common transcript and are located within an intronic region of a potential protein-coding gene [21]. It is likely that they also derive from a common transcript in the chicken, although this has not yet been definitively shown, which implies that they may share similar expression patterns. Furthermore, in our hands the miR-182 probe gave the most robust signal by in situ hybridization compared with miR-96 or miR-183 probes, regardless of which stage was examined. This difference in hybridization signal has also been reported in the mouse retina, as the level of miR-182 was highest and miR-96 was lowest [20]. We suspect that we are too close to the detection threshold to always see radial gradients of miR-96 and miR-183 in the BP, but they are probably present nonetheless.

In the BP at S31, HC expression of all 3 miRNAs is confined to a small cluster of cells on the abneural side in the apical (distal) part of the organ. This location correlates with the earliest differentiation of HCs. At a comparable distal location, young HCs are immunopositive for Tuj1 beginning at S29 [42], the antibody HCA (directed against PTPRQ) at S29 [43–45] and HCS-1 by E7 [38]. Parallel staining of miR-182 and HCS-1 at S29 and S30 (n = 4 ears from 2 embryos at each stage, S4 Fig) suggests that HCS-1 expression in immature HCs begins slightly earlier than miR-182. There is weak labeling of a few HCS-1+ cells near the luminal surface of the BP at the apex at S29, whereas miR-182 is not yet detectable in adjacent sections. At stage 30, both HCS-1 and miR-182 are now expressed in the HC layer, although HCS-1 expression extends through a larger number of cross-sections along the BP compared with miR-182. By S31, overt HC differentiation begins with the appearance of immature stereocilia bundles [46]. Thus, robust expression of the miR-183 family in BP HCs is tightly linked to the initiation of HC differentiation.

### Expression of the miR-183 family in the BP at mid to late gestation

**Fig 2:**
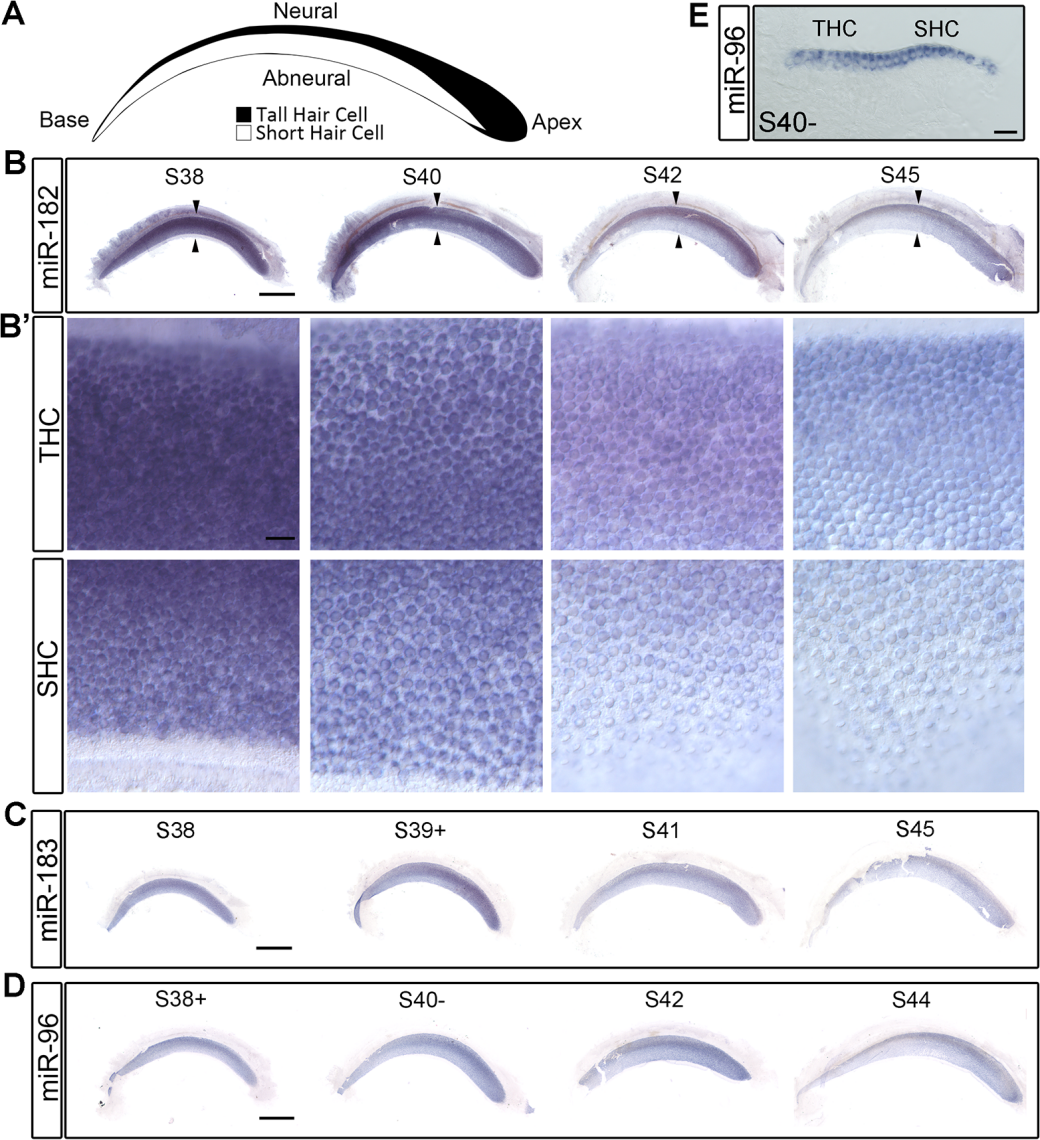
miR-183 family is present in HCs in S38–S45 BPs. (A) The cartoon of THC and SHC distribution in the BP is modified from Tanaka and Smith, 1978 [5]. (B-D) The expression of miR-182, miR-183 and miR-96 in the BPs from S38 to S45. The regions denoted by the arrowheads in B are shown at higher magnification in B’. (E) Cross section through S40- BP after in situ hybridization of miR-96 confirmed that miR-96 is only present in HCs, not in SCs. Scale bar in B-D equals 0.5mm. Scale bar in B’ and E equals 20μm.

To evaluate BP expression patterns at later embryonic stages, in situ hybridization of partially dissected cochlear ducts was performed at daily intervals from E12–E18, encompassing S38–S45. Expression of the miR-183 family was specific to HCs throughout these ages (Fig 2B–2E), and began to decline after S40. At S38–S40, there was no apparent expression gradient along the longitudinal axis of the BP with any of the probes. Moreover, miR-182 failed to show a longitudinal gradient at even earlier stages S35–S36 (n = 2 embryos). At S42–S45, an expression gradient from apex (higher) to base (lower) was apparent, which mimics the directional gradient reported in the mouse cochlea of the adult, but not the neonate [24]. Since HCs in the BP are more densely packed in the apex as compared to the base, we confirmed that the longitudinal intensity differences observed from whole mounts were also detectable at the single cell level after the BPs were first cryosectioned and subjected to in situ hybridization (S5 Fig). A longitudinal miRNA gradient may be significant for regulating gene expression, as microarray expression data revealed that predicted targets for both miR-182 and miR-96 were enriched in the basal (proximal) BP at P0, where the miRNA levels are lower [13].

In addition to this longitudinal gradient, the hybridization signals appeared stronger on the more-densely packed neural half of the BPs when viewed from the surface (Fig 2B–2D). However, in this case, subsequent sectioning of whole mounts failed to reveal qualitative differences in the intensity of HC labeling across the radial axis (Fig 2E). For further confirmation, independent specimens were sectioned first and then subjected to in situ hybridization. These also did not show systematic differences between THCs and SHCs (S5 Fig). The whole mounts were prepared after S38 when the morphological divergence between THCs and SHCs is becoming apparent. This difference likely creates the appearance of a radial gradient due to superimposition of the signal across the elongated, more densely packed THCs on the neural side of the BP. Our conclusion for the chicken BP stands in contrast to data from the mouse, where qualitative differences in the expression of the miR-183 family were observed for IHCs versus OHCs in both whole mounts and sections [24, 25].

### Functional testing of pGFP-183F expression vector *in vitro*

**Fig 3:**
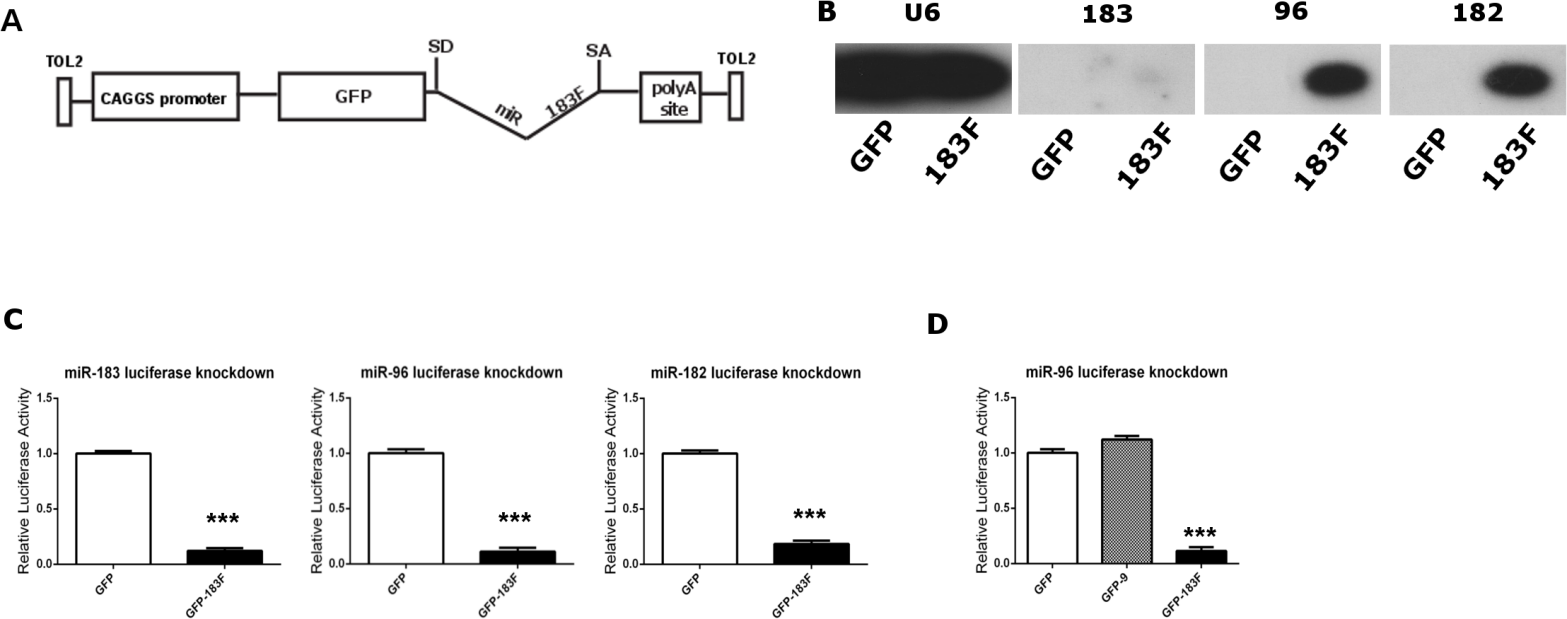
The bifunctional pT2K-CAG-EGFP-183F plasmid expresses functional members of the miR-183 family *in vitro*. (A) Schematic of pT2K-CAG-EGFP-183F. (B) Cells transfected with pGFP-183F show detectable expression of miR-183 family members. Compared to the controls (cells transfected with pGFP), HEK 293T cells transfected with pGFP-183F display expression of mature miR-183, -96, and -182. U6 served as the loading control. (C) miRNAs produced from pGFP-183F bind to their complementary targets to decrease luciferase activity. The miRNA reporters, composed of two complementary miRNA binding sites (to either miR-183, miR-96, or miR-182) housed downstream of the *Renilla* luciferase gene, were co-transfected into DF-1 cells with pGFP-183F or pGFP. When pGFP-183F was included in the transfection, the appropriate miRNA reporter showed a significant decrease in luminescence compared to control wells (DF-1 cells co-transfected with pGFP and the appropriate miRNA reporter). (D) Reporters respond specifically to their miRNAs of interest. The luciferase activity of the miR-96 reporter is significantly decreased when co-transfected with the pGFP-183F vector compared to the control (cells containing pGFP and the reporter). However, no statistically significant change in luminescence is seen in cells co-transfected with the reporter and a comparable expression vector for miR-9 (pGFP-9) compared to the control. Each bar represents mean (±standard error) for each group. Each experiment was replicated at least three times. *** *p* < 0.0001.

To examine the role of the miR-183 family in HC development, we employed an overexpression strategy modified from a design reported previously [30]. Genomic sequences from the murine miR-183/96 and miR-182 loci were fused and placed within an artificial intron, which is located downstream of the EGFP reporter gene. This design exploits a common miRNA-processing pathway that recognizes miRNA hairpins located within introns [47]. This design allows simultaneous delivery and coordinated expression of EGFP and mature miRNAs. Integration into the host cell genome is necessary to ensure long-term expression of the transgenes, and Tol2-mediated transposition was used for this purpose [48]. The GFP-miRNA-intron expression cassette was moved into the pT2K-CAG-EGFP plasmid [29] (abbreviated as pGFP) to create pT2K-CAG-EGFP-183F (abbreviated as pGFP-183F; Fig 3A), which provides flanking Tol2 ends. When the Tol2 vectors are co-expressed with the Tol2 transposase encoded by pCAG-T2TP, the sequence between the Tol2 ends will be randomly inserted into the genome of transfected cells. Once present in the cell, the CAG promoter will drive expression of either GFP (for the control vector pGFP) or a GFP-miRNA transcript (for pGFP-183F). In the latter case, the artificial intron containing primary miRNA hairpin sequences will be excised and further processed into mature miRNAs while the spliced GFP mRNA will be exported to the cytoplasm for translation.

To ensure that mature members of the miR-183 family were produced from the miRNA expression plasmid, HEK293T cells were transfected with pGFP-183F, and their lysates were analyzed using Northern blots. Small RNAs were collected from these cells, probed for the mature miRNA sequence of each miR-183 family member and compared to control samples (pGFP-transfected cells). None of the control samples exhibited any expression of the miRNAs whereas all three members were present in pGFP-183F-transfected cells (Fig 3B). However, miR-183 was significantly weaker on Northern blots than the other two miRNAs.

While Northern blots confirmed that the miRNA-183 family members were expressed from an artificial intron housed within a Tol2 construct, we sought evidence that each mature miRNA was processed and functional in chicken cells by using an *in vitro* dual luciferase assay. As described previously [30], luciferase reporters were constructed for each miRNA by placing two sequences complementary to the miRNA of interest (i.e., miRNA binding sites) downstream of the *Renilla* luciferase gene of the psi-CHECK2 vector (Promega). These reporters were then individually co-transfected with either pGFP-183F or the control vector, pGFP, into a chicken fibroblast cell line. The luciferase luminescence ratio (*Renilla* luminescence over firefly luminescence, controlled for transfection efficiency) of the miRNA-overexpressing wells showed at least an 82% decrease compared to the control for each miRNA reporter tested (Fig 3C; p < 0.0001 for each). The reductions obtained were 89% knockdown for the miR-96 reporter, 82% knockdown for the miR-182 reporter and 88% knockdown for the miR-183 reporter. Thus, despite large differences in miRNA expression levels detected by Northern blots of transfected human HEK-293T cells (Fig 3B), the three miRNAs are roughly equivalent in their bioactivity when assayed in transfected chicken fibroblasts (Fig 3C). Controls for reporter specificity were conducted using an unrelated miRNA, miR-9 housed in the pGFP backbone (pGFP-9), and the miR-96 luciferase reporter. Luminescence only decreased when the miR-96 reporter was co-transfected with pGFP-183F, but not pGFP-9 (Fig 3D).

### Overexpression of the miR-183 family *in vivo*

**Fig 4:**
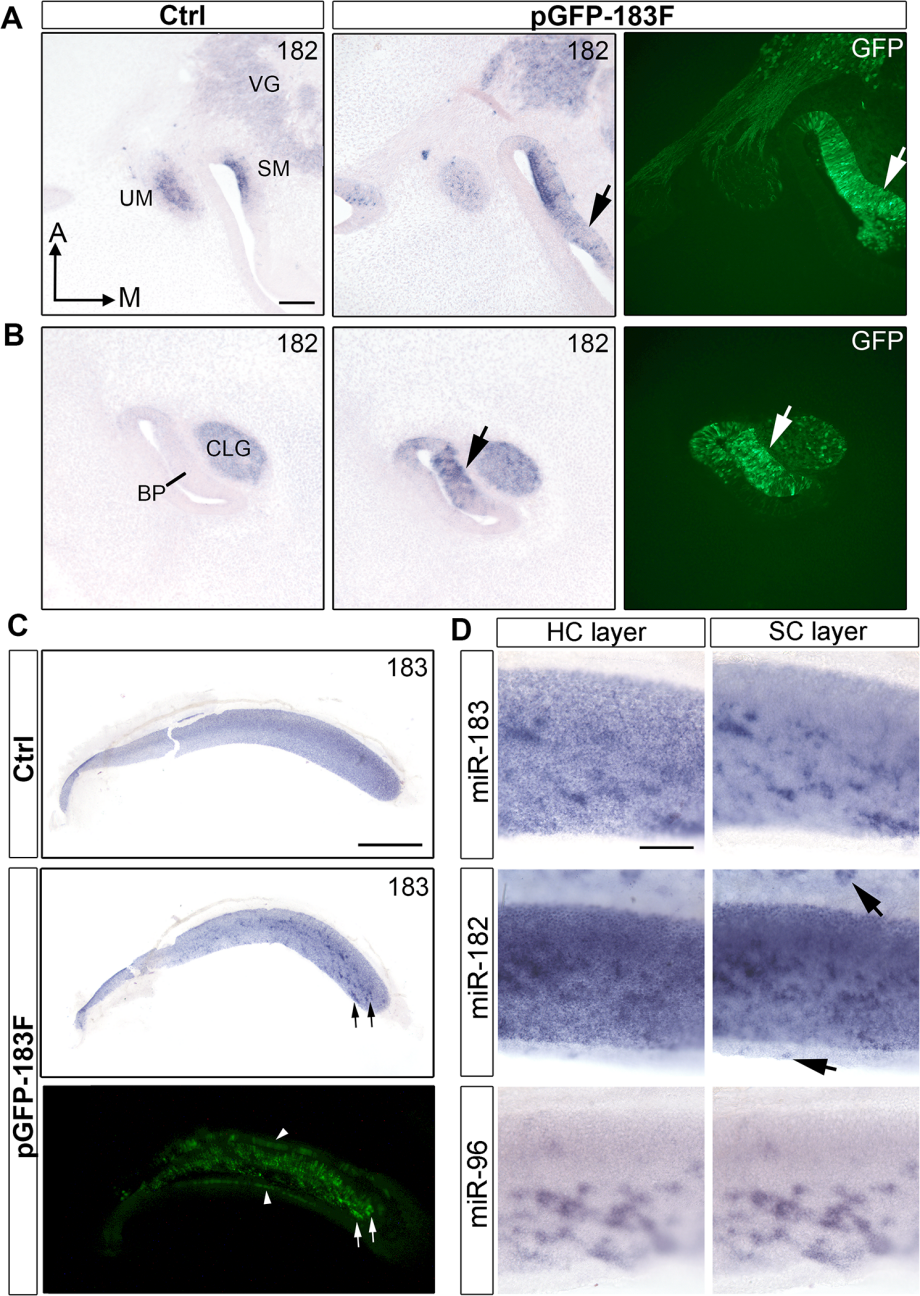
Ectopic expression of miR-183 family is confirmed *in vivo* at S31 and S40. (A, B) miR-182 expression in left control and right pGFP-183F-transfected ears of a S31 embryo that was electroporated at S17. Immunostaining of GFP from adjacent sections through the right BP is shown in the right column. (A) Sections through the maculae show ectopic miR-182 signal in the right ear that overlaps with GFP immunolabeling adjacent to the saccular macula (arrows). Also, foci of stronger miR-182 signal in the vestibular ganglion correspond to regions showing GFP+ cells. (B) Sections through the BPs show GFP expression in (arrows) and adjacent to the sensory region that overlaps with foci of higher miR-182 signal. Ectopic expression is also present in the cochleolagenar ganglion. (C) miR-183 expression shown by in situ hybridization in left control and right pGFP-183F-electroporated BPs at S40 (electroporated at S11+), with GFP fluorescence for comparison in the right ear. Arrows point to examples where GFP+ cells superimpose with a higher intensity of signal for miR-183. Note the presence of GFP in the non-sensory epithelial cells (arrowheads) on both sides of the BP. These transfected cells are not visible in the image taken after in situ hybridization because these epithelial domains were removed for flat mounting. (D) Patches of overexpression in S40 BPs (electroporated at S11+, S12 and S14, respectively) are readily apparent for miR-183, miR-182 and miR-96. Separate images with focus on the HC layer versus SC layer are presented. Arrows point to ectopic expression in non-sensory epithelial cells. Scale bar in A and D equal 100μm. Scale bar in C equals 0.5mm.

Once the functionality of the miRNAs processed from the pGFP-183F construct was confirmed *in vitro*, pGFP-183F and pT2TP were injected and then electroporated into the right otic cup (S11–12) or otocyst (S15–S16) of chicken embryos. The ectopic expression of the miR-183 family was observed at S26 (S6 Fig), before the majority of HCs in the inner ear have exited the cell cycle [40]. In situ hybridization and GFP-immunostaining of adjacent sections at S31 confirmed that the regions showing ectopic miRNA expression co-localized with GFP (Fig 4A and 4B; S7 Fig).

The overexpression of the miR-183 family persisted for two weeks, as HCs were differentiating. Compared with control BPs, pGFP-183F-electroporated BPs showed ectopic expression of the family members in both SCs (Fig 4C and 4D) and non-sensory epithelial cells (Fig 4D) at S40. Although many HCs were obviously GFP+ when viewed in immunostained whole-mounts, we were unable to confirm that the miR-183 family levels exceeded endogenous levels in HCs by whole mount in situ hybridizations of the BP (Fig 4D).

### Ectopic HCs in non-sensory epithelia did not correlate with delivery of a miR-183 family expression vector

**Fig 5:**
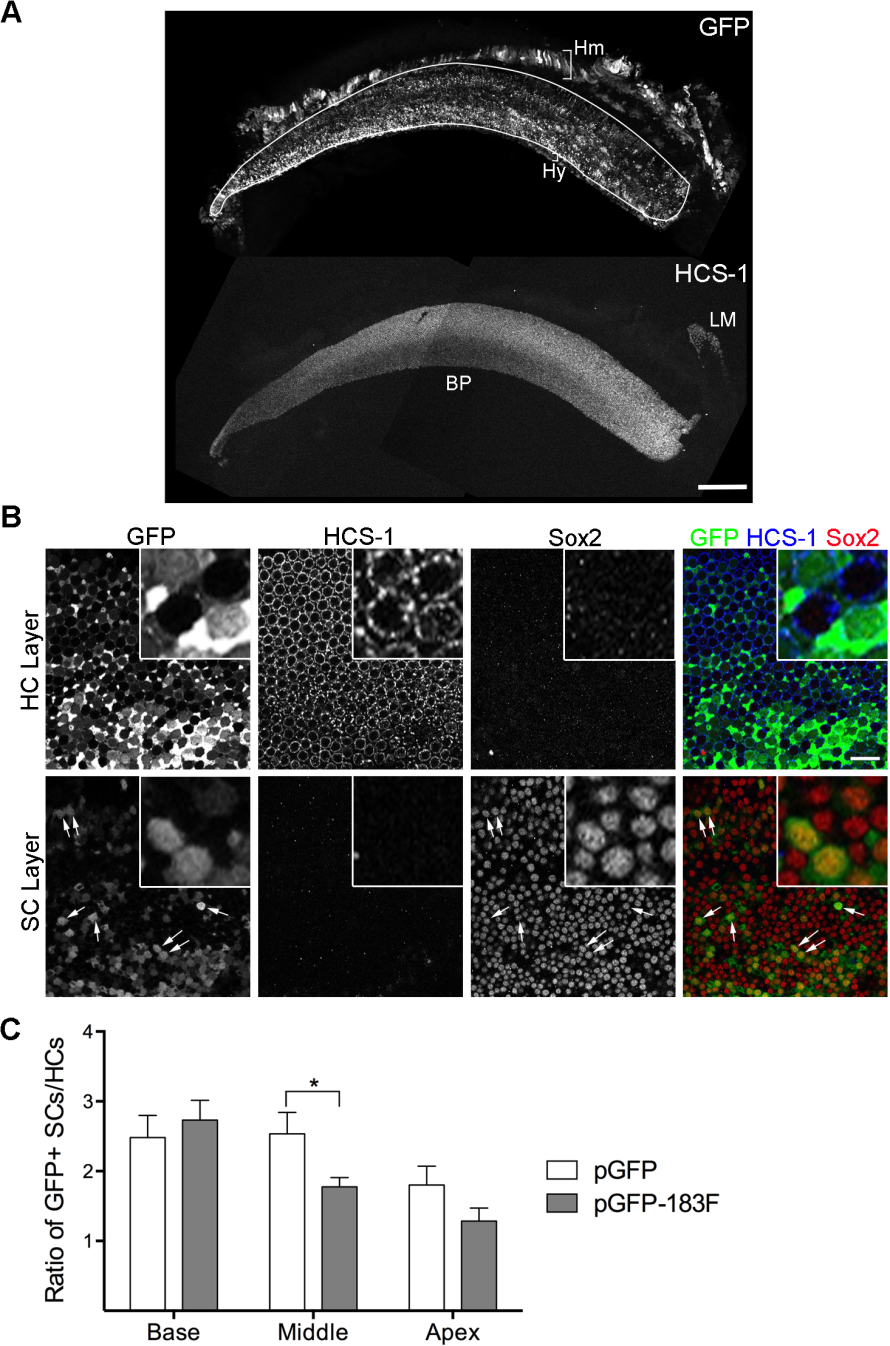
Ectopic expression of miR-183 family can bias progenitor cells towards HC fate. (A) A S38 BP electroporated with pGFP-183F at S11- is immunostained with GFP and HCS-1. HCS-1 staining is present in the BP and the lagena, but not in GFP+ non-sensory epithelia (Hm: homogene cells; Hy: hyaline cells). (B) A S38 BP electroporated with pGFP-183F is stained with antibodies to GFP, HCS-1 and Sox2. Images through the layers of HCs and SCs are shown. Co-localization of Sox2 and HCS-1 is not observed. Arrows are pointing to a few GFP+ SC nuclei. Note that GFP+ SC nuclei have similar Sox2 levels as GFP-negative SC nuclei. Boxed areas are shown with higher magnification. (C) The number of GFP+ SCs and HCs are counted from BPs electroporated with pGFP-183F (n = 9) or pGFP (n = 8) and the ratio of GFP+ SCs/GFP+ HCs is calculated and compared (mean ± SEM). This ratio shows a significant difference (p < 0.05) by multiple t-tests between pGFP-183F and pGFP-electroporated samples only at the middle position along the longitudinal axis of the BP. 2-way ANOVA shows that the ratio is significantly affected by the positions along the BPs. Scale bar in A equals 100μm and in B equals 20μm.

In the zebrafish inner ear, overexpression of miR-96 or miR-182 induced ectopic sensory patches and extra HCs at 26 hpf [26]. Therefore, we posited that overexpression of miR-183 family might also induce non-sensory epithelia to take on a sensory fate in the chicken inner ear. To test this, HCs were labeled with the HCS-1 antibody to detect otoferlin in control and experimental ears. At S26-32 in sectioned samples, no ectopic HC patches were found outside of normal sensory patches in pGFP-183F-electroporated ears (n = 4; S7 Fig). In whole mount BPs from S38–S41, 90% of the transfected right BPs (n = 30/33) did not show ectopic HCs (Fig 5A). However, a total of 5 ectopic sensory patches of various sizes were observed in 3 embryos. These patches were found both superior (neural) and inferior (abneural) to the normal boundaries of the BP, and at various proximal-distal positions. However, their existence was not correlated with pGFP-183F transfection (3 were GFP-negative and 2 were GFP+). In a single embryo, the left control ear had a patch of 4 ectopic HCs located 70% from the base on the neural side, while the right transfected ear had two ectopic patches, both GFP-negative (24 HCs at 70% from the base on the neural side and ~300 HCs at 90% from the base on the abneural side). Of the two GFP+ ectopic patches observed in 2 other electroporated right ears, one (65 HCs) was located at 90% from the base on the abneural side and the other (9 HCs) was located at 30% from the base on the neural side. Thus, while ectopic HCs are occasionally observed in embryos subjected to electroporation, overexpression of the miR-183 family does not appear to increase the likelihood of their appearance, assuming that GFP is a reliable reporter of miRNA overexpression (Fig 4A–4C). Moreover, because we never saw evidence of these ectopic HC patches in non-electroporated embryos, it may be that the electroporation process can perturb otic development on either side of the embryo, perhaps causing small patches of cells to become isolated from the main organ.

### Progenitor cells are biased toward an HC fate by transfection with a miR-183 family expression vector

Since the miR-183 family was ectopically expressed as early as S26, before cells in the BP begin to pull out of division, we asked whether this experimental manipulation would push bipotential progenitor cells toward a HC fate rather than a SC fate. The number of GFP+ HCs and SCs were counted in stage 38–40 BPs transfected with control (pGFP) or experimental (pGFP-183F) plasmids, and the ratio of GFP+ SCs/GFP+ HCs was calculated. This ratio is expected to decrease if pGFP-183F-transfected cells were preferentially assuming a HC fate. Indeed, there was a decline in this ratio in the middle and apical sample areas when comparing the pGFP-183F and pGFP conditions (Fig 5B and 5C). However, the decrease was only statistically significant (p < 0.05, t-test) for the mid-longitudinal BP (Fig 5C). The regional restriction of cell fate changes to the middle of the BP is difficult to explain based on our current knowledge of BP developmental gradients in cell birthdates (basal-to-apical) or cell differentiation (apical-to-basal) [40, 45]. Nonetheless, this finding provides indirect evidence that some of the targets for the miR-183 family may be present at the prosensory stage, and may be modulated by changes in the level of the miR-183 family.

One way in which ectopic miRNAs might promote a HC fate is by repressing target transcripts normally found in progenitor cells or SCs. In the developing cochlea, Sox2 initially marks bipotential progenitor cells and then becomes restricted to SCs as it becomes downregulated in differentiating HCs [49, 50]. Using a luciferase assay, Weston and colleagues found that the 3’UTR of murine *Sox2* mRNA was a target of miR-182 *in vitro* [24], although we did not find a comparable result for the 3’UTR of human *Sox2* mRNA using a luciferase assay [51]. Also, a miRNA target prediction algorithm (TargetScan, version 6.2) applied to the 3’UTR of chicken *Sox2* failed to find predicted binding sites for any of the miR-183 family members. Nonetheless, we asked whether ectopic expression of the miR-183 family decreased the level of Sox2 protein in avian SCs, providing a possible mechanism by which they might be pushed toward a HC fate. Whole-mount BPs from pGFP-183F-transfected embryos were triple-labeled with antibodies to Sox2, GFP antibody and HCS-1 at S40. There was no systematic qualitative difference in the intensity of Sox2 immunolabeling when comparing GFP+ SCs to neighboring GFP-negative SCs within the same sample. Furthermore, cells double-positive for GFP and Sox2 were never co-labeled with HCS-1 (Fig 5B). This suggests that delivery of miR-183 family expression plasmids does not generate a persistent mixed HC-SC phenotype, when assayed with the markers used.

### HC morphologies and HC subtypes are unaltered by transfection with a miR-183 family expression vector

**Fig 6:**
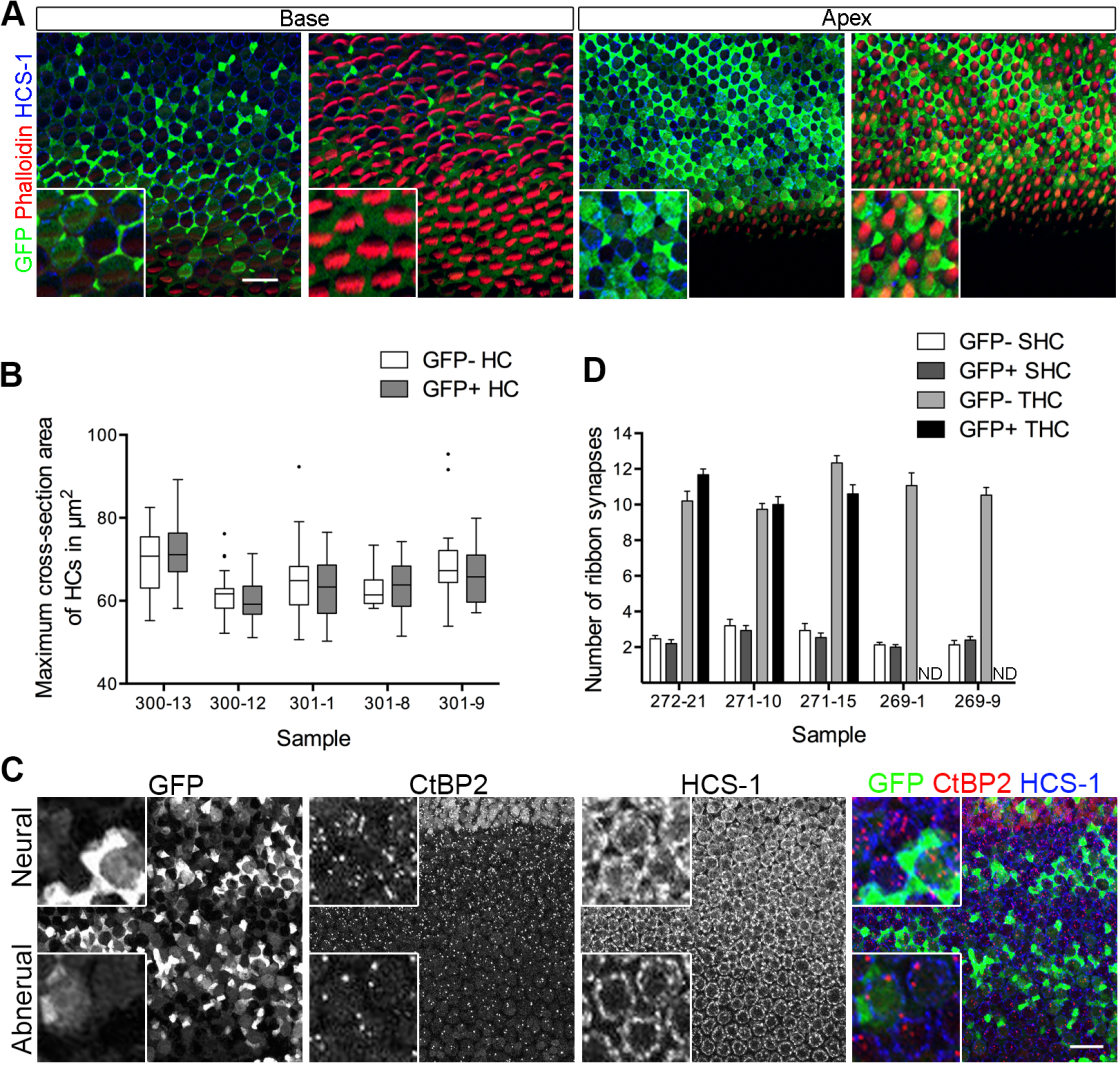
Ectopic expression of miR-183 family does not alter HC fate along and across the BP. (A) A S40- BP electroporated with pGFP-183F at S11 is stained with GFP, phalloidin that labels actin and HC marker HCS-1. Images through the layer of HC bodies and stereocilia bundles at the base and the apex are shown. GFP+ basal HCs have similar cell cross-section areas and similar width of stereocilia bundles compared with their GFP-negative neighboring HCs, thus they do not gain the characteristics of apical HCs. (B) Quantification of maximum cross-section areas of HCs are done in the base of pGFP-183F transfected BPs. Tukey box and whiskers are shown here with outliers shown in dots. 2-way ANOVA showed no significant difference between GFP-negative HCs (GFP-) and GFP+ HCs. (C) A S40- BP electroporated with pGFP-183F at S15 is stained with GFP, CtBP2 that labels ribbon synapses and HCS-1. Maximum intensity projections of the image stack at the base of the BP are shown with neural on top. (D) Numbers of ribbon synapses in GFP+ SHCs, GFP-negative SHCs, GFP-negative THCs and GFP+ THCs from the base of pGFP-183F electroporated BPs (n = 5) are compared (mean ± SEM). The ribbon synapse numbers in GFP+ SHCs and GFP-negative SHCs are not significantly different by 2-way ANOVA analysis. Scale bar in A and C equals 20μm. Boxed areas are shown with high magnification. ND: not determined.

During BP development, the miR-183 family is expressed in a gradient along the longitudinal (tonotopic) axis, with highest levels found at the apex. The longitudinal axis of the BPs shows systematic changes in stereociliary bundle morphologies (shorter and wider at the base) and in the lumenal surface areas of the HCs (larger at the base) [52]. These features were recently used to monitor changes in the longitudinal identity of HCs in response to molecular manipulations of BMPs and retinoids [11, 12]. To ask whether delivery of miR-183 family expression plasmids could affect these morphological parameters, pGFP-183F-transfected BPs were triple-labeled with antibodies to GFP (to identify transfected cells), HCS-1 (to measure HC cross-sectional areas) and phalloidin (to label HC bundles). The presence of GFP was used as an indirect indicator of ectopic miRNA expression. We predicted that increasing the expression of the miR-183 family might cause basal HCs to acquire the characteristics of apical HCs, because the apex is where their expression levels are normally highest. However, no differences were found between GFP+ basal HCs and their GFP-negative neighboring HCs in maximal cross-sectional areas (Fig 6A and 6B). Paradoxically, Weston and colleagues [24] found that the conditional deletion of *Dicer* in mouse cochlear HCs yielded a greater number of genes that were differently expressed between the apex and the base, suggesting that HC miRNAs function to suppress (rather than to promote) cochlear gradients of gene expression.

Prior to the appearance of a longitudinal gradient, we observed an early radial gradient in the expression of the miR-183 family (highest on the neural side) in the prosensory BP. We reasoned that genes responsible for imparting neural versus abneural identity to the prosensory cells might be post-transcriptionally regulated by this miR-183 family expression gradient. Such an early influence on radial identity might be manifested later by the phenotypic differences between THCs and SHCs. Specifically, we speculated that HCs located on the abneural half of the BP, which normally become SHCs, might acquire the morphological phenotypes of THCs if they are forced to overexpress the miR-183 family beginning at the prosensory stage. One of the major differences between THCs and SHCs is that the former HCs make far more ribbon synapses with afferent neurites than the latter. Synapse number per HC can be quantified by immunolabeling with an antibody directed against CtBP2, a marker of ribbon synapses. Focusing on the basal half of the BP, we found that the number of ribbon synapses per HC was not significantly different between GFP+ HCs and their neighboring GFP-negative HCs (Fig 6C and 6D).

We conclude that in the BP, delivery of the miR-183 family expression plasmid does not impact the differentiation of THC versus SHC phenotypes. This result is notable considering that miR-96 plays an important role in HC maturation and survival in the mouse, as revealed by the phenotypes observed in *Dimineundo (Dmdo)* mutant mice [18, 53, 54]. *Dmdo* has been mapped to a point mutation in the seed region of miR-96. In the homozygous condition, this leads to differentiation arrest of IHCs and OHCs after birth. Much of this phenotype can be explained by a decrease in PTPRQ [54]. The differentiation of HCs progresses further in *Dmdo/+* mice, such that IHCs and OHCs are distinguishable postnatally, but their maintenance and survival is compromised by one month of age. These data suggest that HCs are particularly sensitive to the levels of wildtype miR-96, but do not rule out the possibility of a gain-of-function effect in which miR-96^*Dmdo*^ binds to inappropriate targets. Our data from the chick do not support the idea that it is the absolute levels of wildtype miR-96 in nascent HCs that drives their separation into different phenotypes.

There are several technical caveats that might explain why the delivery of a miR-183 family overexpression vector ectopically to the inner ear on E2/E3 did not alter HC phenotypes 10–14 days later. One possibility is that phenotypic changes may require longer survival times than were used here. The longitudinal gradient of miR-183 family was observed after S42. However, due to poor survivals and a developmental lag of 1–3 days among electroporated embryos, most of those processed were staged as S40 and younger. The second possibility is that the levels of the three miRNAs were not ectopically increased in HCs *in vivo*, despite the obvious overexpression of GFP in transfected cells. We could not detect miRNA overexpression in HCs by in situ hybridization, due to strong endogenous HC expression and insufficient resolution between HCs and the apical processes of transfected SCs. And yet, *in vitro* luciferase assays and Northern blots revealed that functional, mature miRNAs were generated from the expression vector, and ectopic miRNAs were clearly detectable in SCs and non-sensory domains *in vivo*. Thus, the possibility must be considered that miRNA levels are more tightly regulated in HCs than in other cells of the inner ear, like in photoreceptors in mouse retina [55]. Perhaps HCs cannot persistently overexpress the miR-183 family due to the presence of an unknown regulatory pathway that can negatively feedback on the transcription, processing or stability of these miRNAs when their levels are artificially raised. Finally, we consider the possibility that overexpression levels were present but were too modest to sufficiently reduce the miRNA targets and thereby produce downstream effects. In general, many miRNAs only moderately modulate target levels, rather than serving as on/off switches for target protein expression [56, 57].

Until now, deliberate experimental manipulation of the miR-183 family members has mainly relied on knockdown or knockout approaches [21, 58]. Gene knockdowns or knockouts in the retina have revealed that down-regulation or loss of the miR-183 family leads to progressive degeneration of photoreceptors and increased susceptibility to light damage [21, 58]. There are two studies using gain-of-function approaches of the miR-183 family. One study introduced double-stranded miRNA mimics into zebrafish embryos at single-cell stage [26]. The other study was performed in mouse retina [59]. AAVs encoding short hairpin RNAs that resemble pre-miR-183 and pre-miR-182 were injected into the retinas of DGCR8-conditional knockout mice in which miRNA biogenesis was blocked, leading to re-expression of miR-183 and miR-182 in Cre-expressing cone photoreceptors and prevention of the loss of outer segments and cone opsins [59]. It is interesting to consider that the levels of the miR-183 family are physiologically relevant to retinal function, since they are down-regulated during dark adaptation and up-regulated in light-adapted retina [55]. In the chicken cochlea, we used a gain-of-function approach to seek evidence that level differences in this gene family may also play an important developmental role in this sensory organ. We found that manipulating the levels of the miR-183 family can mildly influence the HC-SC fate decision. However, using our experimental approach, ectopic expression of the miR-183 family was insufficient to redirect the differentiation of HCs towards specific radial or longitudinal phenotypes.

## Supporting Information

S1 Fig Sequence comparison of the miR-183 family members among human, mouse, chicken and zebrafish. miR-96 is conserved among the four species, while miR-182 and miR-183 show differences in the last few nucleotides at their 3’ ends. Here, hsa-miR-96/182/183, mmu-miR-96/182/183 and dre-miR-182 stand for hsa-miR-96/182/183-5p, mmu-miR-96/182/183-5p and dre-miR-182-5p, respectively. Seed regions, nucleotides 2–7, are highlighted.
(TIF)

S2 Fig The expression of miR-183 in the inner ear at S28 and S31. (A-C) Horizontal sections through the inner ear. The sections in A-C and the sections in Fig 1A come from one S28 embryo. (D-F) Transverse sections through the inner ear. At S28, miR-183 is weakly expressed in the anterior and posterior cristae (A), the saccular macula and the vestibular ganglion (B), but is not detected in the cochlear duct (C). At S31, miR-183 is strongly expressed in HCs of all the vestibular organs (D-E) and the apical part of the BP (D-F). It is detected in neurons of both the vestibular ganglion (D) and the cochleolagenar ganglion (E, F). Abbreviations: A, anterior; AC, anterior crista; BP, basilar papilla; CG, cochleolagenar ganglion; D, dorsal; LC: lateral crista; LM: lagena macula; M, medial; PC, posterior crista; SM, saccular macula; UM, utricular macula; VG, vestibular ganglion. Scale bar equals 100 μm.
(TIF)

S3 Fig The expression of miR-96 in the inner ear at S28 and S32. (A-C) Horizontal sections through the inner ear at S28. D-J: Horizontal sections through the inner ear at S32. At S28, expression of miR-96 is detected in the cristae (A, B), the utricular and saccular maculae (B), and the vestibular and cochleolagenar ganglia (B, C). At S32, miR-96 expression is robust in HCs of all three cristae (D, E, G) and the three maculae (H, J). Two patterns of expression are observed in the BP: a weak radial gradient in the basal organ at the prosensory stage (bracket), and HC-associated expression in the apical organ (I). Weak expression is maintained in the ganglia at S32 (F, H). Abbreviations are the same as S2 Fig. Scale bar equals 100 μm.
(TIF)

S4 Fig Parallel staining of miR-182 and HCS-1 at S29–S31. (A) Adjacent sections across the ear at S31 are stained with miR-182 or HCS-1. Note that both miR-182 and HCS-1 staining is weaker in the BP compared with the vestibular organs at this stage. Arrows point to examples of cytoplasmic tails of HCs toward the basal side of the epithelium. (B) Adjacent sections across the BP at S29 and S30. Weak HCS-1+ HCs are observed at the apex of the BP at S29, but miR-182 is not detected in adjacent sections. Both HCS-1 and miR-182 are detectable at S30. Abbreviations are the same as S2 Fig. Scale bar equals 50 μm.
(TIF)

S5 Fig Expression of miR-182 in cross sections through the basilar papilla at S43. Section in situ hybridization through the BP allows comparisons of signal intensities along and across the organ. The percentage depicts the section position from the extreme base (0%) to the apex (100%). Within each section, there was no obvious qualitative difference in miR-182 signal intensity between THCs on the neural side and SHCs on the abneural side. However, a longitudinal gradient from base (weaker) to apex (stronger) is apparent in both types of HC. Scale bar equals 50 μm.
(TIF)

S6 Fig Ectopic miR-96 expression can be observed two days after plasmid electroporation. Alternate horizontal sections through the inner ears of one embryo at S26, following electroporation of the right ear with pGFP-183F and pT2TP at S15, are shown. Alternate sections are processed for in situ hybridization of miR-96 (A, B, D, E) or immunostained with an anti-GFP antibody and AlexFluor-488 secondary antibody to enhance the detection of GFP (C, F). At this stage, the control ear shows weak expression of miR-96 in vestibular organs and the vestibular ganglion (A) but no expression in the BP (D). The electroporated ear shows ectopic expression of miR-96 (arrows in B, E) in the inner ear epithelia that corresponds to GFP immunolabeling in nearby sections (arrows in C, F). Abbreviations are the same as S2 Fig Scale bar equals 100 μm.
(TIF)

S7 Fig Similar ectopic expression is observed for both miR-182 and miR-96 four days after plasmid electroporation. Horizontal sections through one embryo at S31, following electroporation of pGFP-183F and pT2TP into the right ear at S17, are shown. Alternate sections are processed for detection of miR-182 or miR-96 by in situ hybridization, or immunostained for GFP and HCS-1 (to detect HCs) as indicated on the panels. There is ectopic expression of miR-182 and miR-96 in the sensory and non-sensory epithelia and also in the vestibular and cochleolagenar ganglion neurons depicted in B, E, I and L. The signals detected by in situ hybridization are comparable to those shown by immunolabeling for GFP in nearby sections. However, there are no ectopic HCs observed in transfected non-sensory domains (compare arrow in G to a similar location in E, and compare arrow in M to a similar location in J). Although there were a few HCS-1+ cells in the mesenchyme in both F and G, similar staining was also seen in untransfected specimens. Abbreviations are the same as S2 Fig. Scale bar equals 100μm. Note that images in A-C and H-J are also shown in Fig 5.
(TIF)

S8 Fig All supporting information figures. S1–S8 Figs combined as a Compressed/ZIP file Archive.
(PDF)

S1 Text IACUC waiver. Letter documenting the waiver of protocol approval for use of chicken embryos.
(PDF)
