# Supplementary figures and images for: Expression and Misexpression of the miR-183 Family in the Developing Hearing Organ of the Chicken
Source: PLoS One. 2015 Jul 15;10(7):e0132796. doi: 10.1371/journal.pone.0132796 (PMC4503353; doi:10.1371/journal.pone.0132796)

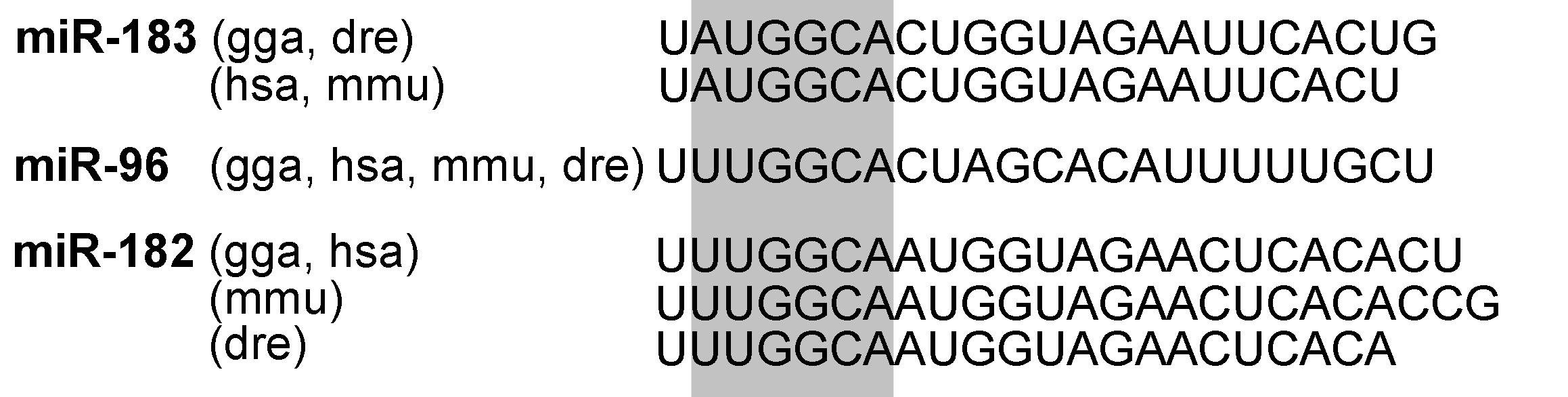

Supplement: S1 Fig — miR-96 is conserved among the four species, while miR-182 and miR-183 show differences in the last few nucleotides at their 3’ ends. Here, hsa-miR-96/182/183, mmu-miR-96/182/183 and dre-miR-182 stand for hsa-miR-96/182/183-5p, mmu-miR-96/182/183-5p and dre-miR-182-5p, respectively. Seed regions, nucleotides 2–7, are highlighted. (TIF) [file pone.0132796.s001.tif]

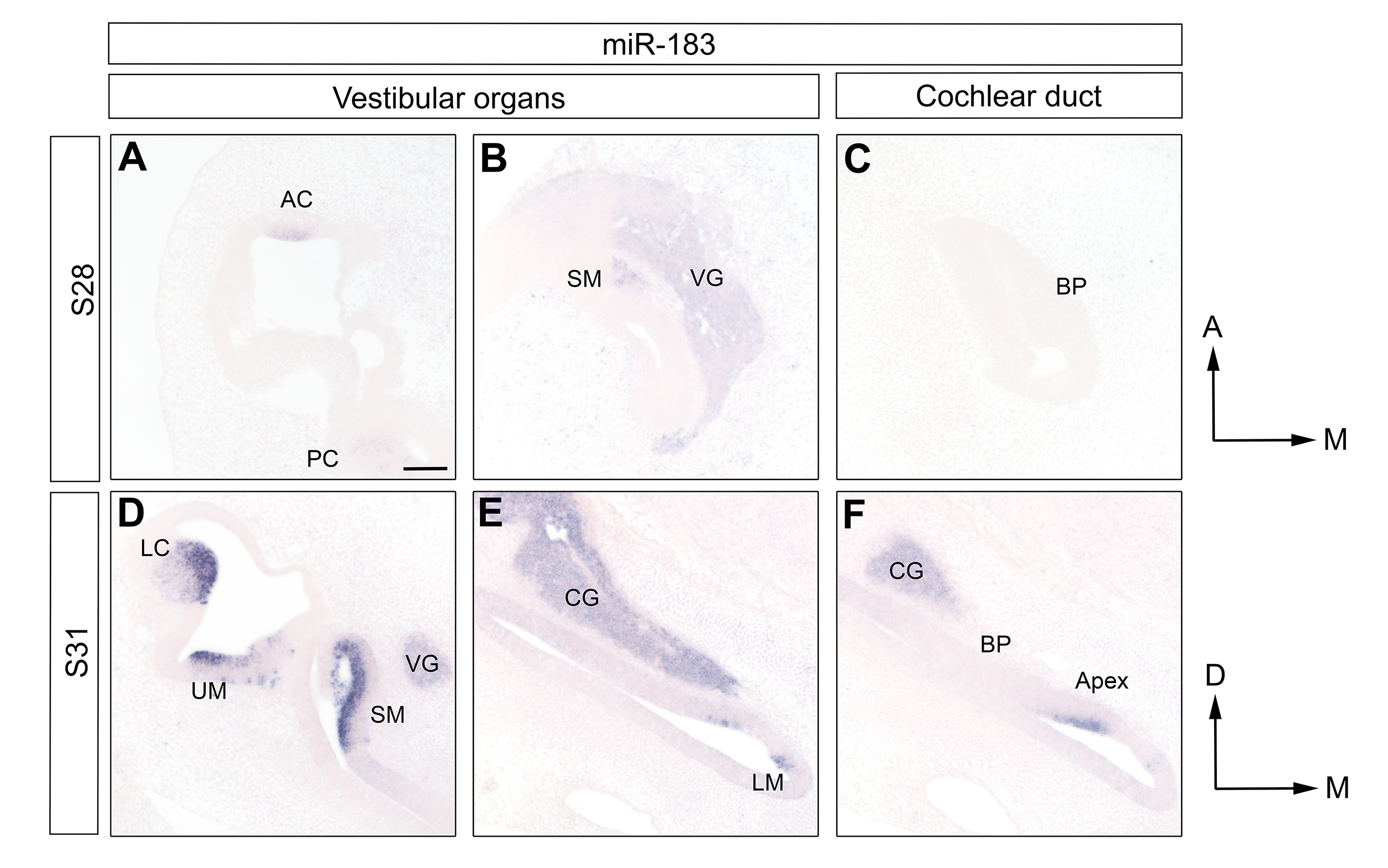

Supplement: S2 Fig — (A-C) Horizontal sections through the inner ear. The sections in A-C and the sections in Fig 1A come from one S28 embryo. (D-F) Transverse sections through the inner ear. At S28, miR-183 is weakly expressed in the anterior and posterior cristae (A), the saccular macula and the vestibular ganglion (B), but is not detected in the cochlear duct (C). At S31, miR-183 is strongly expressed in HCs of all the vestibular organs (D-E) and the apical part of the BP (D-F). It is detected in neurons of both the vestibular ganglion (D) and the cochleolagenar ganglion (E, F). Abbreviations: A, anterior; AC, anterior crista; BP, basilar papilla; CG, cochleolagenar ganglion; D, dorsal; LC: lateral crista; LM: lagena macula; M, medial; PC, posterior crista; SM, saccular macula; UM, utricular macula; VG, vestibular ganglion. Scale bar equals 100 μm. (TIF) [file pone.0132796.s002.tif]

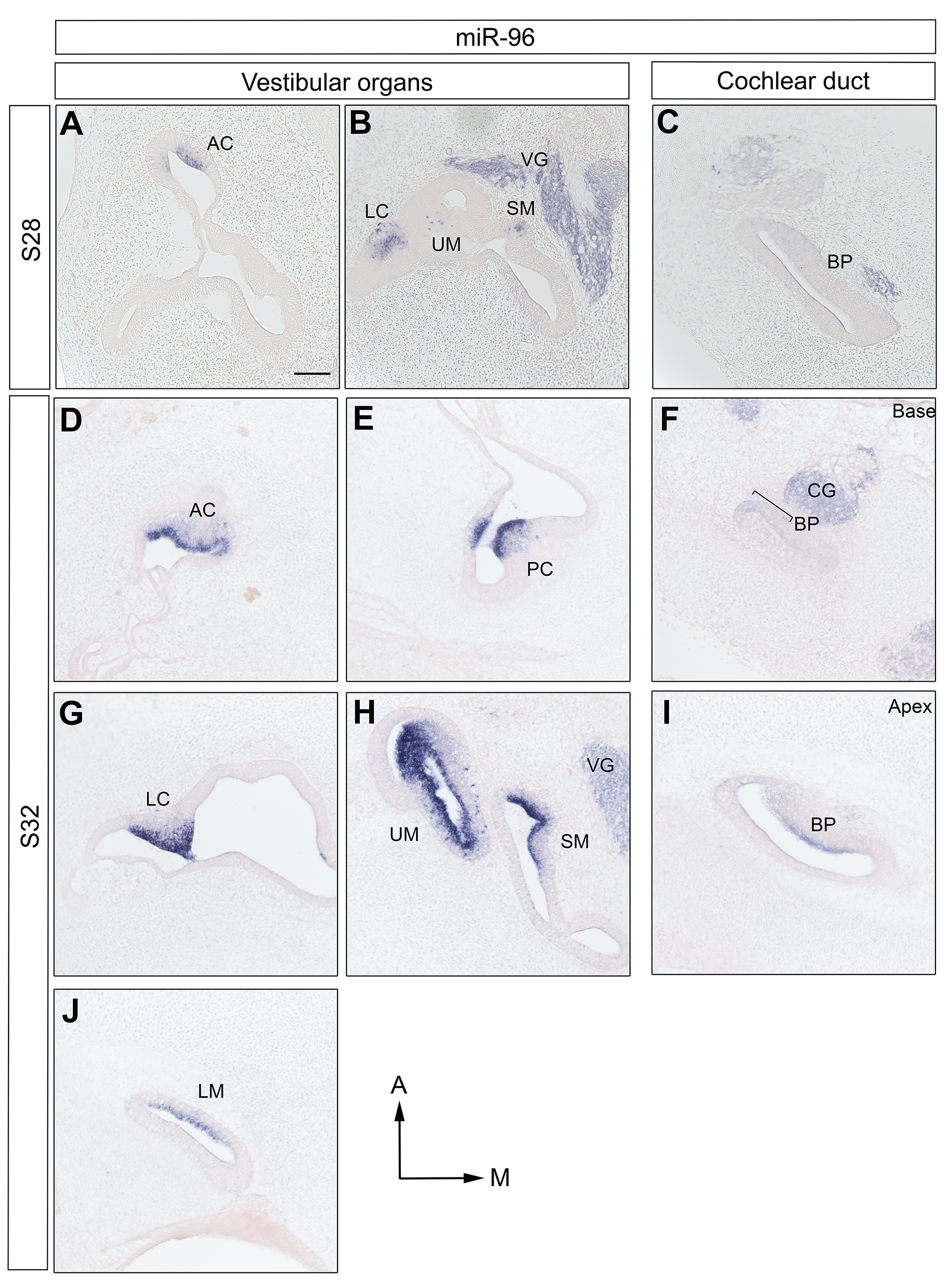

Supplement: S3 Fig — (A-C) Horizontal sections through the inner ear at S28. D-J: Horizontal sections through the inner ear at S32. At S28, expression of miR-96 is detected in the cristae (A, B), the utricular and saccular maculae (B), and the vestibular and cochleolagenar ganglia (B, C). At S32, miR-96 expression is robust in HCs of all three cristae (D, E, G) and the three maculae (H, J). Two patterns of expression are observed in the BP: a weak radial gradient in the basal organ at the prosensory stage (bracket), and HC-associated expression in the apical organ (I). Weak expression is maintained in the ganglia at S32 (F, H). Abbreviations are the same as S2 Fig. Scale bar equals 100 μm. (TIF) [file pone.0132796.s003.tif]

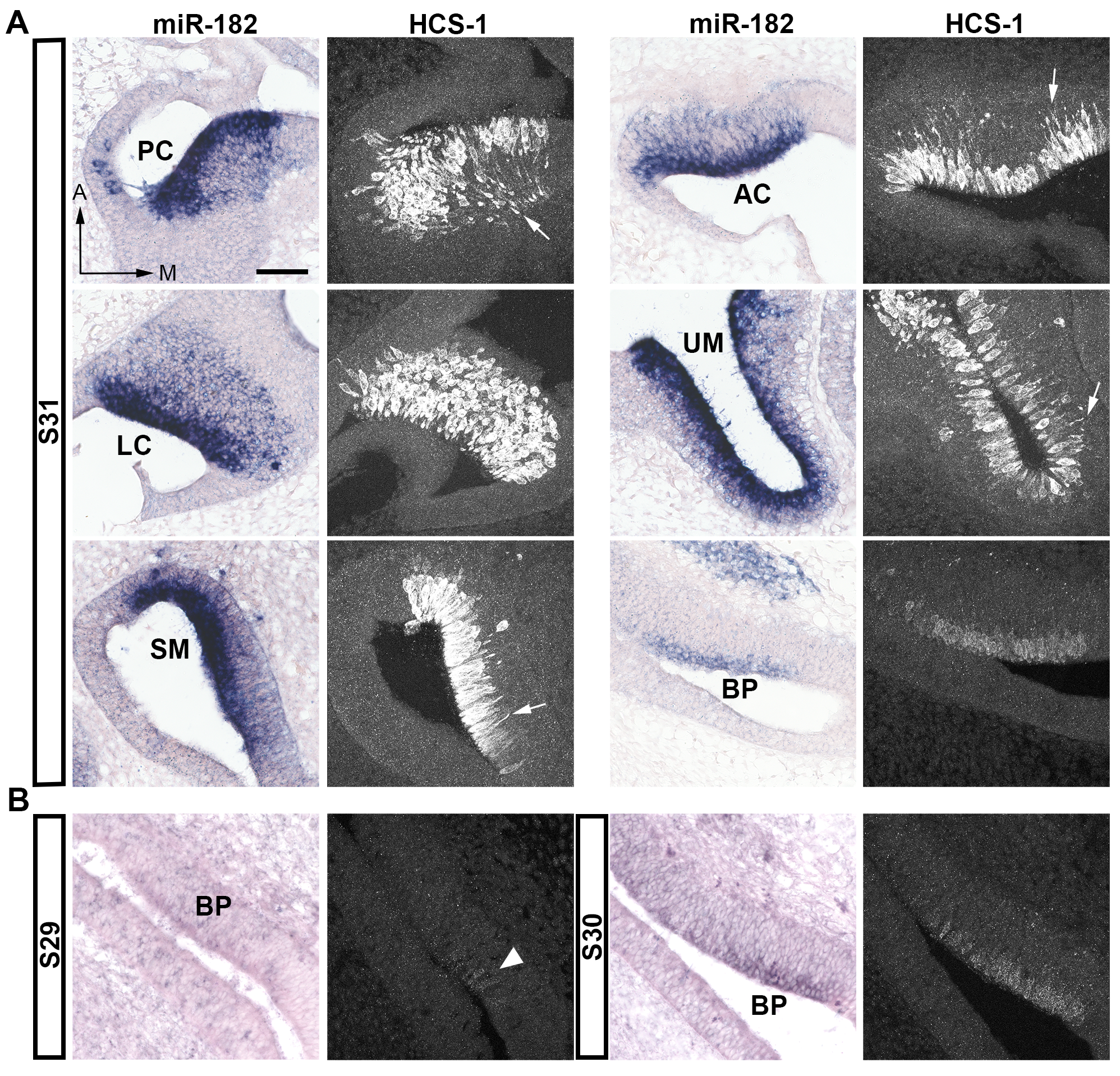

Supplement: S4 Fig — (A) Adjacent sections across the ear at S31 are stained with miR-182 or HCS-1. Note that both miR-182 and HCS-1 staining is weaker in the BP compared with the vestibular organs at this stage. Arrows point to examples of cytoplasmic tails of HCs toward the basal side of the epithelium. (B) Adjacent sections across the BP at S29 and S30. Weak HCS-1+ HCs are observed at the apex of the BP at S29, but miR-182 is not detected in adjacent sections. Both HCS-1 and miR-182 are detectable at S30. Abbreviations are the same as S2 Fig. Scale bar equals 50 μm. (TIF) [file pone.0132796.s004.tif]

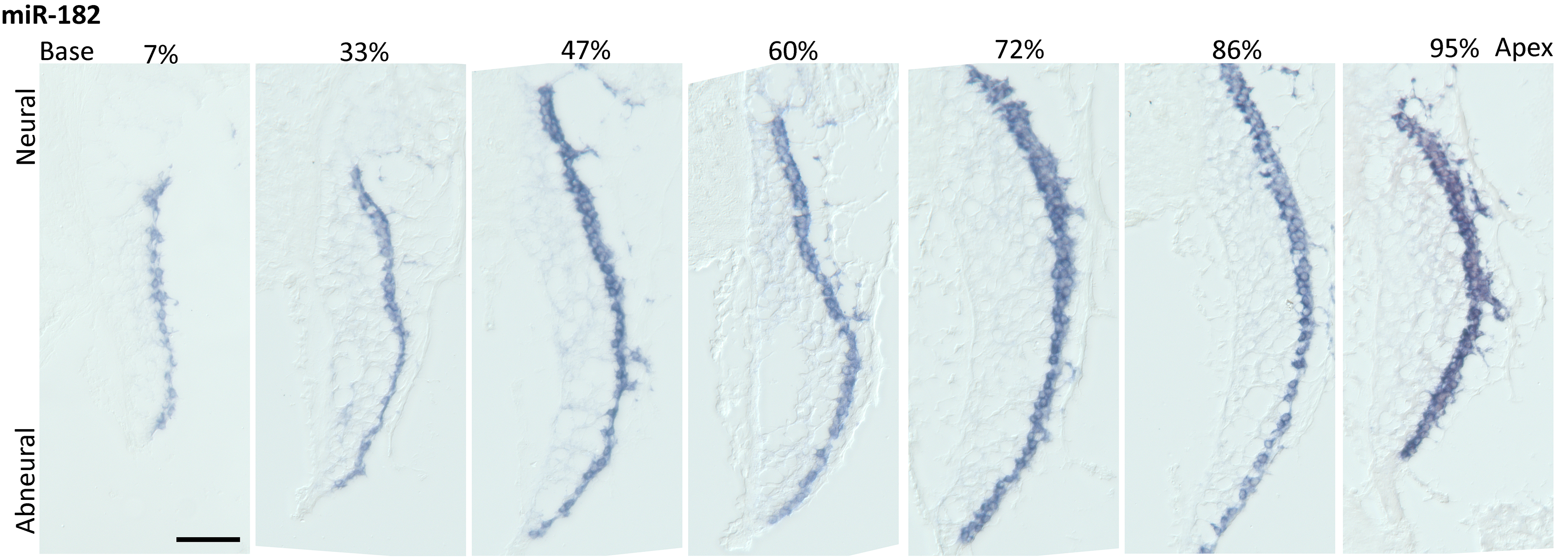

Supplement: S5 Fig — Section in situ hybridization through the BP allows comparisons of signal intensities along and across the organ. The percentage depicts the section position from the extreme base (0%) to the apex (100%). Within each section, there was no obvious qualitative difference in miR-182 signal intensity between THCs on the neural side and SHCs on the abneural side. However, a longitudinal gradient from base (weaker) to apex (stronger) is apparent in both types of HC. Scale bar equals 50 μm. (TIF) [file pone.0132796.s005.tif]

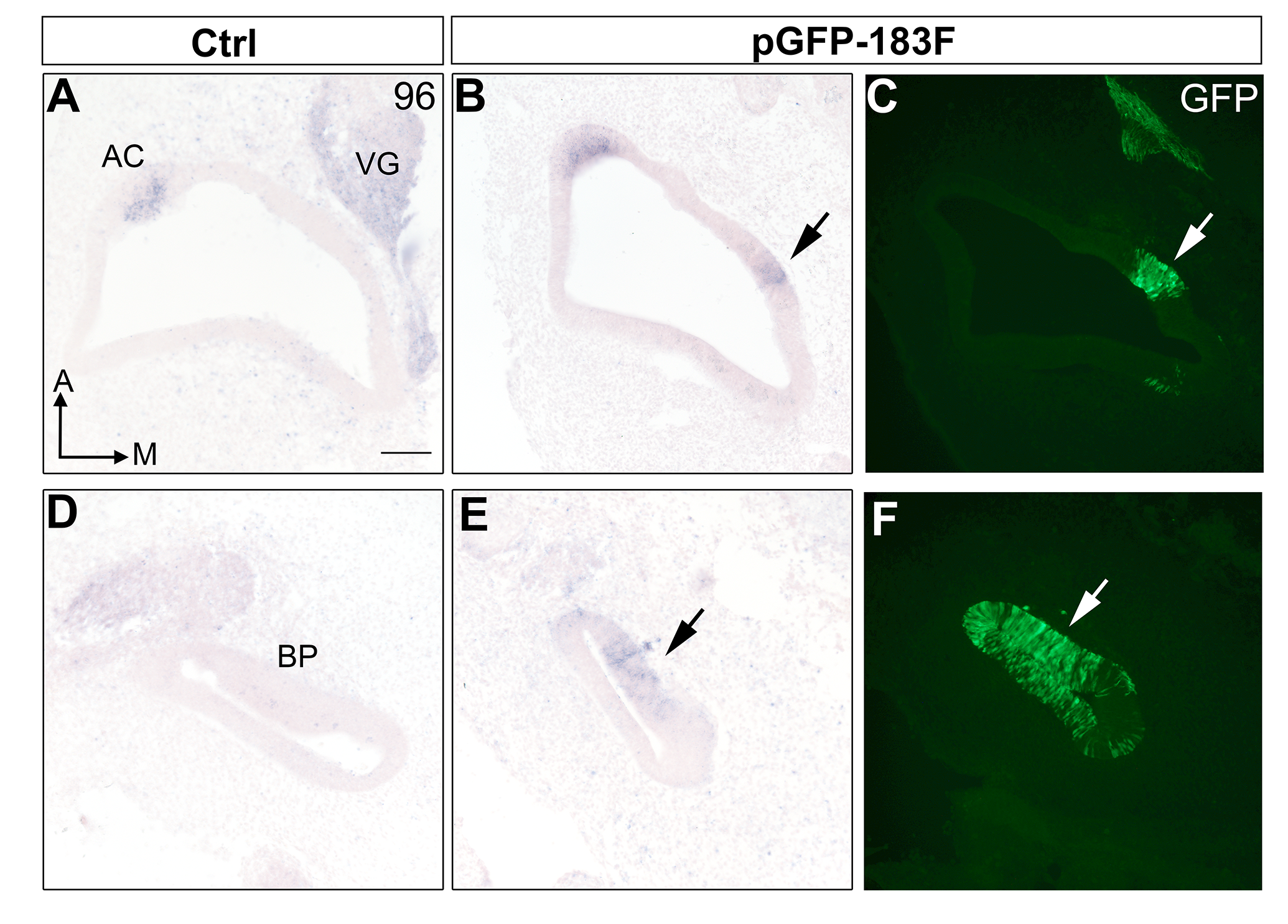

Supplement: S6 Fig — Alternate horizontal sections through the inner ears of one embryo at S26, following electroporation of the right ear with pGFP-183F and pT2TP at S15, are shown. Alternate sections are processed for in situ hybridization of miR-96 (A, B, D, E) or immunostained with an anti-GFP antibody and AlexFluor-488 secondary antibody to enhance the detection of GFP (C, F). At this stage, the control ear shows weak expression of miR-96 in vestibular organs and the vestibular ganglion (A) but no expression in the BP (D). The electroporated ear shows ectopic expression of miR-96 (arrows in B, E) in the inner ear epithelia that corresponds to GFP immunolabeling in nearby sections (arrows in C, F). Abbreviations are the same as S2 Fig Scale bar equals 100 μm. (TIF) [file pone.0132796.s006.tif]

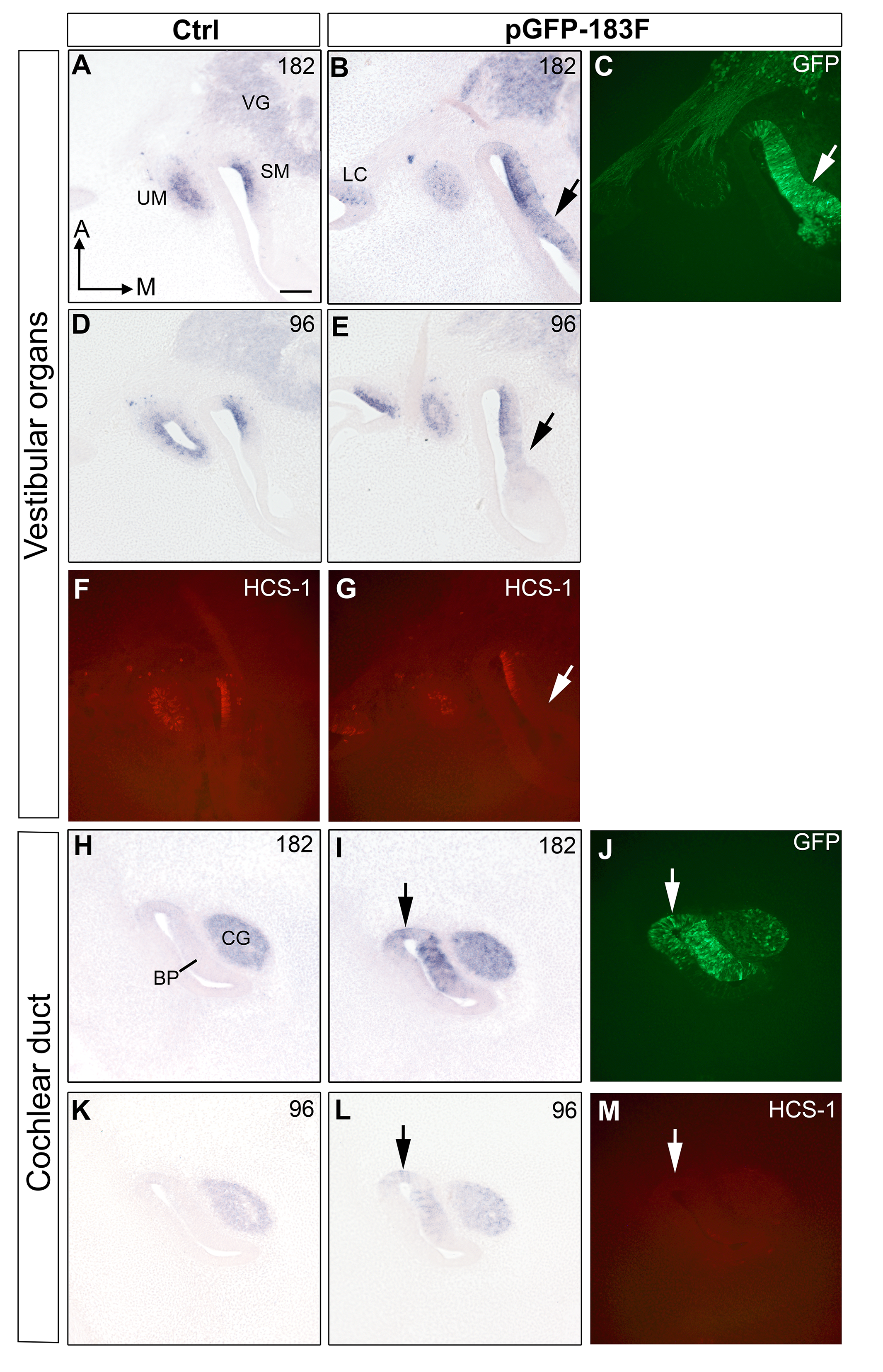

Supplement: S7 Fig — Horizontal sections through one embryo at S31, following electroporation of pGFP-183F and pT2TP into the right ear at S17, are shown. Alternate sections are processed for detection of miR-182 or miR-96 by in situ hybridization, or immunostained for GFP and HCS-1 (to detect HCs) as indicated on the panels. There is ectopic expression of miR-182 and miR-96 in the sensory and non-sensory epithelia and also in the vestibular and cochleolagenar ganglion neurons depicted in B, E, I and L. The signals detected by in situ hybridization are comparable to those shown by immunolabeling for GFP in nearby sections. However, there are no ectopic HCs observed in transfected non-sensory domains (compare arrow in G to a similar location in E, and compare arrow in M to a similar location in J). Although there were a few HCS-1+ cells in the mesenchyme in both F and G, similar staining was also seen in untransfected specimens. Abbreviations are the same as S2 Fig. Scale bar equals 100μm. Note that images in A-C and H-J are also shown in Fig 5. (TIF) [file pone.0132796.s007.tif]
