# Supplementary material for: Expression and Misexpression of the miR-183 Family in the Developing Hearing Organ of the Chicken
Source: PLoS One. 2015 Jul 15;10(7):e0132796. doi: 10.1371/journal.pone.0132796 (PMC4503353; doi:10.1371/journal.pone.0132796)
Supplement: S8 Fig — S1–S8 Figs combined as a Compressed/ZIP file Archive. (PDF) [file pone.0132796.s008.pdf]

**miR-183**	(gga, dre)	UAUGGCACUGGUAGAAUUCACUG
	(hsa, mmu)	UAUGGCACUGGUAGAAUUCACU
**miR-96**	(gga, hsa, mmu, dre)	UUUGGCACUAGCACAUUUUUGCU
**miR-182**	(gga, hsa)	UUUGGCAAUGGUAGAACUCACACU
	(mmu)	UUUGGCAAUGGUAGAACUCACACCG
	(dre)	UUUGGCAAUGGUAGAACUCACA

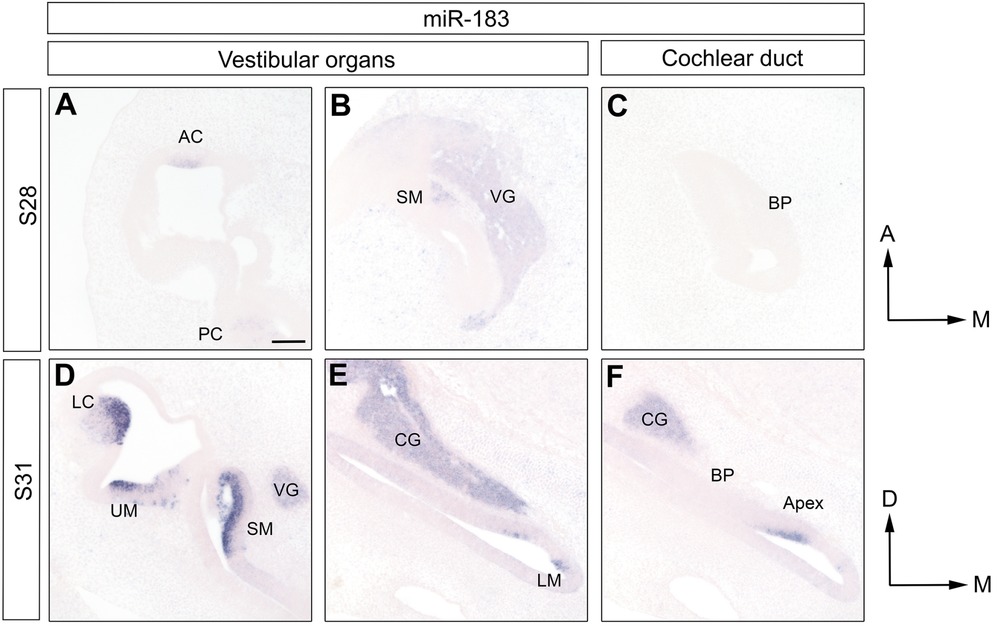
miR-183
Vestibular organs
Cochlear duct
S28
S31
A
AC
PC
B
SM
VG
C
BP
D
LC
UM
SM
VG
E
CG
LM
F
CG
BP
Apex
A
M
D
M

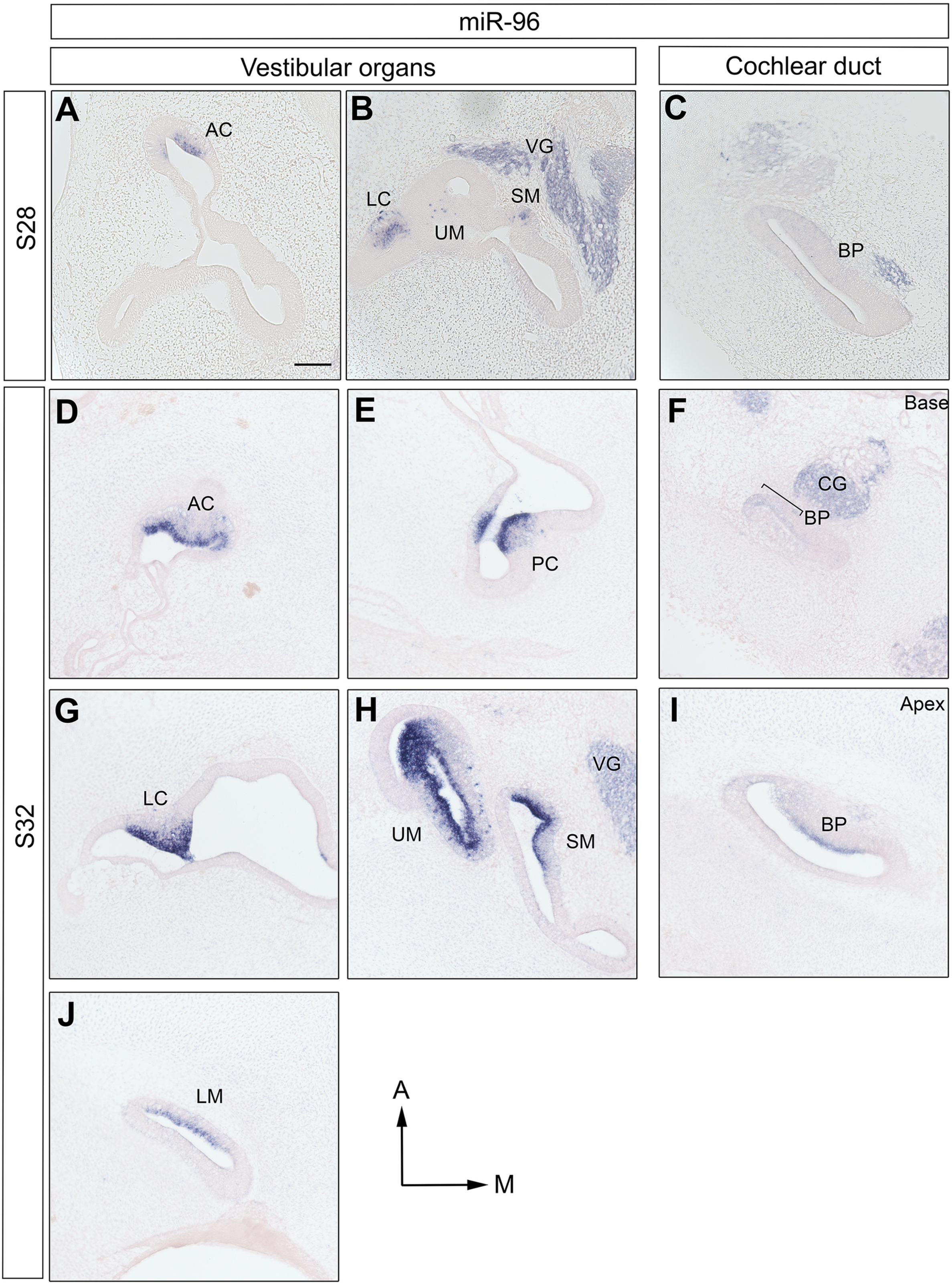

miR-96
Vestibular organs
Cochlear duct
S28
S32
A
AC
B
VG
LC
SM
UM
C
BP
D
AC
E
PC
F
Base
CG
BP
G
LC
H
UM
VG
SM
I
Apex
BP
J
LM
A
M

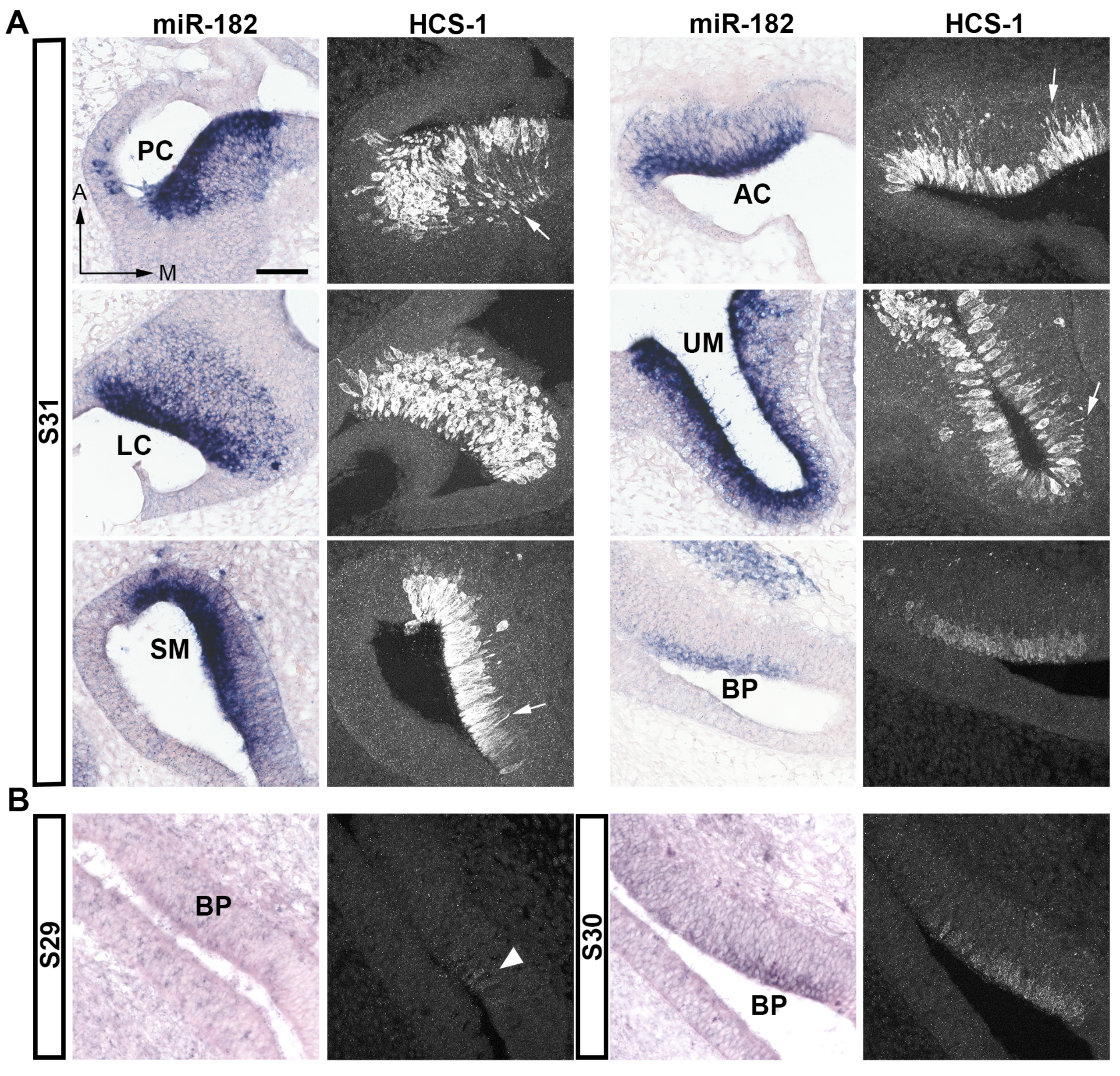
A
miR-182
HCS-1
miR-182
HCS-1
S31
PC
A
M
AC
LC
UM
SM
BP
B
S29
BP
S30
BP

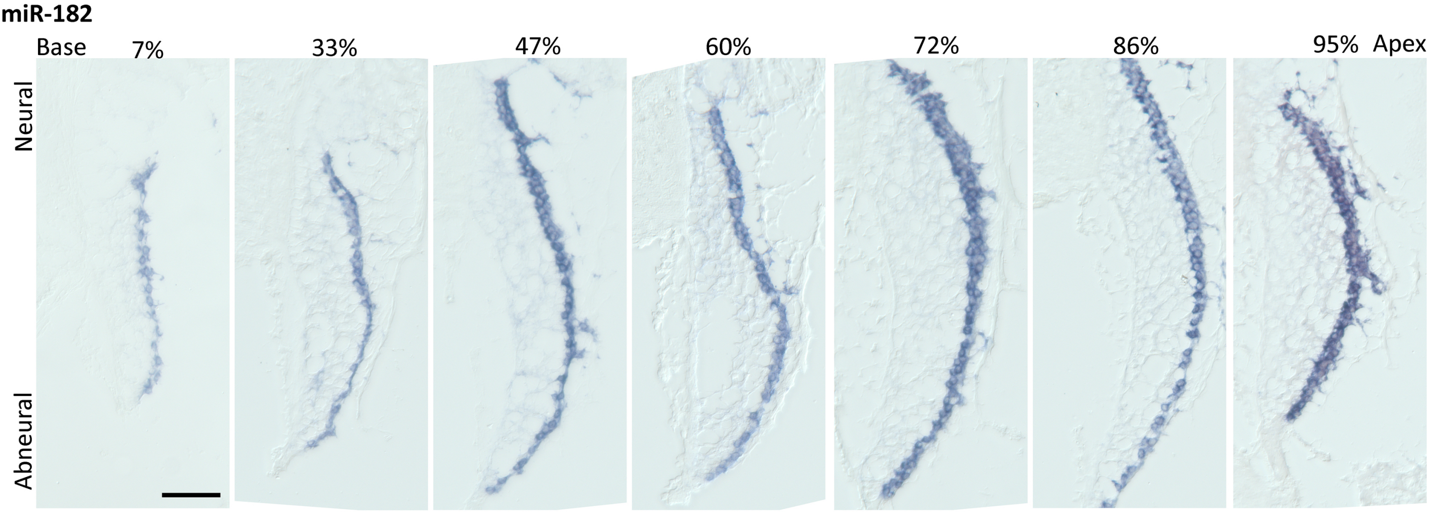

miR-182
Base
7%
33%
47%
60%
72%
86%
95%
Apex
Neural
Abneural

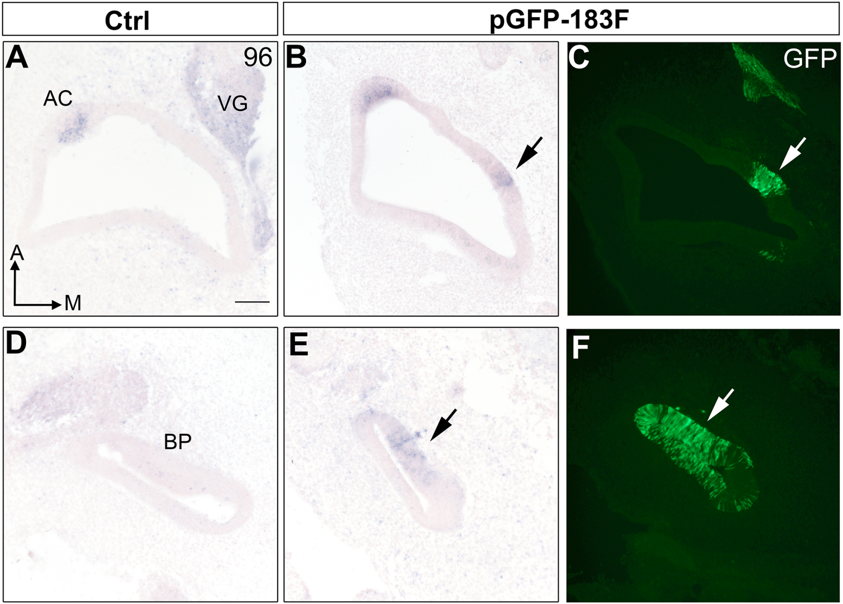
Ctrl
pGFP-183F
A
96
AC
VG
A
M
B
C
GFP
D
BP
E
F

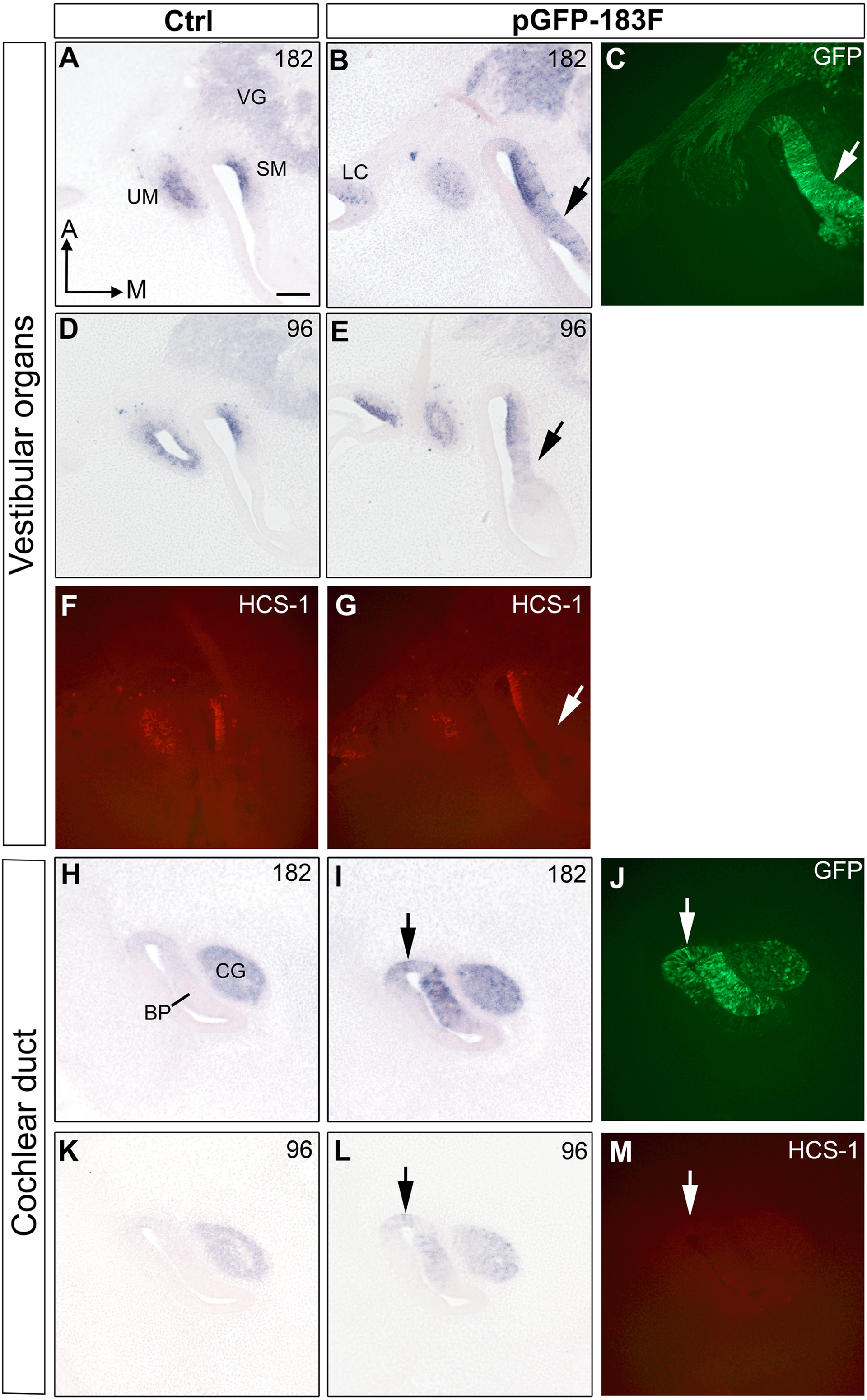
Ctrl
pGFP-183F
Vestibular organs
Cochlear duct
A
182
VG
SM
UM
A
M
B
182
LC
C
GFP
D
96
E
96
F
HCS-1
G
HCS-1
H
182
CG
BP
I
182
J
GFP
K
96
L
96
M
HCS-1
