# Supplementary material for: Expression and Misexpression of the miR-183 Family in the Developing Hearing Organ of the Chicken
Source: PLoS One. 2015 Jul 15;10(7):e0132796. doi: 10.1371/journal.pone.0132796 (PMC4503353; doi:10.1371/journal.pone.0132796)
Supplement: S1 Text — Letter documenting the waiver of protocol approval for use of chicken embryos. (PDF) [file pone.0132796.s009.pdf]

PURDUE
UNIVERSITY

Purdue Animal Care and Use Committee

February 23, 2015

PLOS ONE Journal

To Whom It May Concern:

This letter is to certify that Prof. Donna Fekete did not need approval from Purdue's Institutional Animal Care and Use Committee (IACUC) for her work with chicken embryos. Prof. Fekete used embryos up to 18 days developed that were immediately euthanized by decapitation. Purdue's IACUC does not require an approved protocol for chickens until day 21 when they hatch into a live animal.

Please feel free to contact this office should you need further information or assistance.

Sincerely,

Lisa Snider

Lisa D. Snider
IACUC Administrator

410 South University Street, VAHF Bldg. • West Lafayette, IN 47907
(765) 494-7206 • Fax: (765) 496-2415 • E-mail: ldsnider@purdue.edu
